# Genomic Diversity among Drug Sensitive and Multidrug Resistant Isolates of *Mycobacterium tuberculosis* with Identical DNA Fingerprints

**DOI:** 10.1371/journal.pone.0007407

**Published:** 2009-10-12

**Authors:** Stefan Niemann, Claudio U. Köser, Sebastien Gagneux, Claudia Plinke, Susanne Homolka, Helen Bignell, Richard J. Carter, R. Keira Cheetham, Anthony Cox, Niall A. Gormley, Paula Kokko-Gonzales, Lisa J. Murray, Roberto Rigatti, Vincent P. Smith, Felix P. M. Arends, Helen S. Cox, Geoff Smith, John A. C. Archer

**Affiliations:** 1 Molecular Mycobacteriology, Research Center Borstel, Borstel, Germany; 2 Department of Genetics, University of Cambridge, Cambridge, United Kingdom; 3 Division of Mycobacterial Research, MRC National Institute for Medical Research, Mill Hill, London, United Kingdom; 4 Illumina Cambridge Limited, Chesterford Research Park, Little Chesterford, Essex, United Kingdom; 5 Burnet Institute for Medical Research and Public Health, Melbourne, Australia; University of Hyderabad, India

## Abstract

**Background:**

*Mycobacterium tuberculosis* complex (MTBC), the causative agent of tuberculosis (TB), is characterized by low sequence diversity making this bacterium one of the classical examples of a genetically monomorphic pathogen. Because of this limited DNA sequence variation, routine genotyping of clinical MTBC isolates for epidemiological purposes relies on highly discriminatory DNA fingerprinting methods based on mobile and repetitive genetic elements. According to the standard view, isolates exhibiting the same fingerprinting pattern are considered direct progeny of the same bacterial clone, and most likely reflect ongoing transmission or disease relapse within individual patients.

**Methodology/Principal Findings:**

Here we further investigated this assumption and used massively parallel whole-genome sequencing to compare one drug-susceptible (K-1) and one multidrug resistant (MDR) isolate (K-2) of a rapidly spreading *M. tuberculosis* Beijing genotype clone from a high incidence region (Karakalpakstan, Uzbekistan). Both isolates shared the same IS*6110* RFLP pattern and the same allele at 23 out of 24 MIRU-VNTR loci.

We generated 23.9 million (K-1) and 33.0 million (K-2) paired 50 bp purity filtered reads corresponding to a mean coverage of 483.5 fold and 656.1 fold respectively. Compared with the laboratory strain H37Rv both Beijing isolates shared 1,209 SNPs. The two Beijing isolates differed by 130 SNPs and one large deletion. The susceptible isolate had 55 specific SNPs, while the MDR variant had 75 specific SNPs, including the five known resistance-conferring mutations.

**Conclusions:**

Our results suggest that *M. tuberculosis* isolates exhibiting identical DNA fingerprinting patterns can harbour substantial genomic diversity. Because this heterogeneity is not captured by traditional genotyping of MTBC, some aspects of the transmission dynamics of tuberculosis could be missed or misinterpreted. Furthermore, a valid differentiation between disease relapse and exogenous reinfection might be impossible using standard genotyping tools if the overall diversity of circulating clones is limited. These findings have important implications for clinical trials of new anti-tuberculosis drugs.

## Introduction

Tuberculosis (TB) is primarily a symptom of poverty and inequality [1], [2], which accounts for its uninterrupted prevalence in most parts of the world [3]. This disparity in TB burden is further exacerbated by the widespread emergence of antibiotic resistance [4]. In Eastern Europe, all of these factors coincide. In Karakalpakstan (Uzbekistan) multi-drug resistant (MDR) *Mycobacterium tuberculosis* complex (Mtb) strains infect up to 22% of patients never treated and up to 60% of those previously treated [3], [4], [5]. Therefore the most effective antibiotics with the fewest side-effects, isoniazid (INH) and rifampicin (RMP), must, in MDR TB, be replaced by less-effective, more expensive, and more toxic substitutes.

In addition to the socio-economic, environmental, and host genetic determinants [6], strain genetic diversity in Mtb appears to play a more important role than previously believed [7], [8], [9], [10]. Mtb comprises several phylogenetic lineages, some of which have been shown to significantly impact progression from infection to active tuberculosis as well as disease manifestation [11], [12], [13]. In Karakalpakstan and other parts of Eastern Europe, the so-called Beijing lineage of Mtb is mostly responsible for the ongoing MDR epidemic [14]. Some members of this strain lineage have been reported to be hypervirulent in animal models [15], and others have been associated with large outbreaks and extensive transmission of MDR TB in various regions of the world [7], [16], [17]. These findings contradict previous assumptions that resistant Mtb strains in general have a reduced fitness. The fact that MDR variants can even have an enhanced fitness when compared with susceptible progenitor strains, points to the phenomenon of compensatory evolution that can reverse adverse effects on bacterial fitness [18], [19].

Even though genetic diversity appears to influence virulence and immunogenicity of strain of different phylogenetic lineages [9], the overall level of sequence variation in Mtb is low and Mtb is considered to represent the “upper limit of what might be considered genetically monomorphic” [20]. Whole genome comparisons of *M. tuberculosis* and *M. bovis*, another species of the *M. tuberculosis* complex, revealed a maximum diversity of just 2,500 SNPs [21]. *M. tuberculosis* isolates of the same clone or strain (e.g. characterised by IS*6110* genotyping) are generally considered to be identical and belong to the same outbreak or recent chain of transmission [22]. However, the real level of genomic diversity in rapidly expanding clones from high incidence settings has not been assessed.

**Figure 1:**
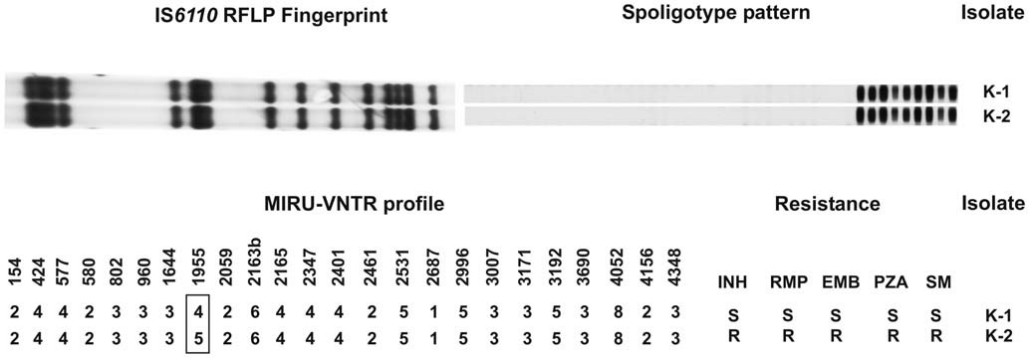
Genotyping and drug resistance data of the strains analysed. All traditional DNA fingerprints for both isolates were isogenic, with the exception of the MIRU-VNTR locus 1955. K-2 was resistant to all five first line antibiotics (S (sensitive), R (resistant)).

To address this question, we have applied massively paralleled whole-genome sequencing [23] to two clinical isolates from Karakalpakstan. These isolates were virtually identical with respect to all standard TB genotyping markers, including IS*6110* RFLP, which is the most discriminating test of all standard molecular TB typing tools in use today [22] (Figure 1). The two isolates, K-1 and K-2, differed in only 1 of 24 Mycobacterial Interspersed Repetitive Units. However, whilst K-1 was fully drug-susceptible, K-2 was resistant to all first line drugs (INH, RMP, ethambutol (EMB), pyrazinamide (PZA) and streptomycin (SM)). Both isolates were part of a large cluster of closely related organisms termed the Beijing K-family that encompasses the dominant, expanding Mtb variant in the region [unpublished results,14].

## Results

### Genome coverage

We generated 23.9 million (K-1) and 33.0 million (K-2) paired 50 bp purity filtered reads corresponding to a mean coverage of 483.5 fold and 656.1 fold respectively which we compared to the genome of the *M. tuberculosis* reference strain H37Rv (ATCC 27294) [24]. To differentiate true differences from sequencing errors in the original H37Rv reference sequence (File S1), we also generated 2.7 Gb of sequence data from strain H37Rv (26.7 million paired 50 bp reads, 538.5-fold coverage). The fraction of the H37Rv genome that could not be covered (approximately 2.0%) comprised DNA with a GC content >80% and repeat regions (such as IS*6110* elements) whose sequence could not be uniquely aligned to the reference. This fraction increased to 4.8% for K-1 and 3.4% for K-2 to exclude any areas for which no valid base was called due to insertion or deletions. All the genetic changes we identified are correlated with the existing scientific literature and listed in detail in the Supplementary Information (File S1). In addition to a tabular output of our results (File S2) a flexible, graphical representation using Artemis [25] is provided (Files S3-S6) to allow an exploration of these changes in their genomic context.

### Errors in reference genome

**Figure 2:**
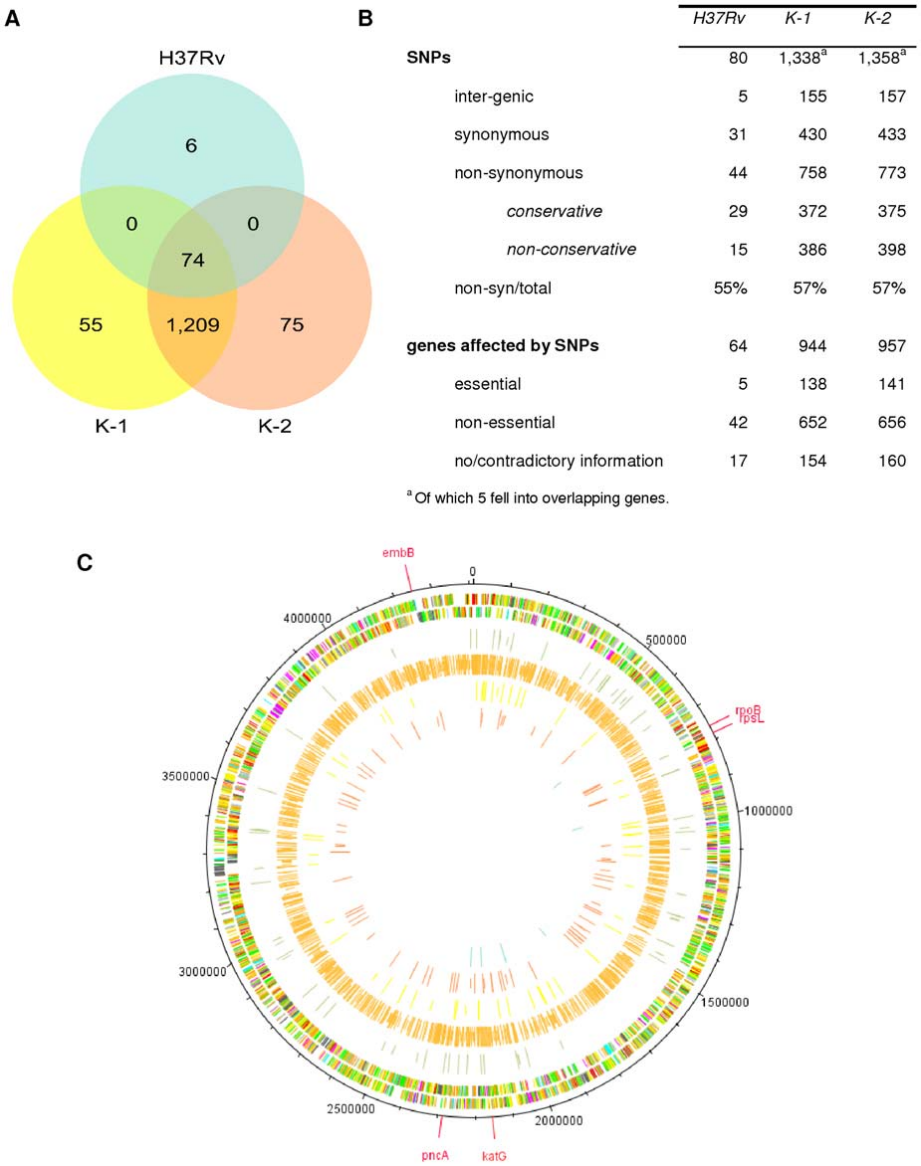
Overview of the genome data obtained. A, Venn diagram showing the SNP distribution between the three genomes under investigation (H37Rv, K-1, and K-2) relative to the published H37Rv sequence. The 75 K-2 specific SNPs encompassed the 5 resistance conferring SNPs (Table 2). 44 of the 74 SNPs shared in the three genomes were found to be errors in the H37Rv reference (File S1). B, Summary of nature and location of SNPs detected in this study. C, Circular plot of H37Rv reference genome prepared with DNAPlotter [46]. The two outer-most circles show the genes on the forward and reverse strand respectively with the annotation and colour coding derived from TubercuList [47]. The remaining 5 internal circles correspond to the 5 filled subsection of the Venn diagram in Figure 2A with identical colour coding (from third to inner-most circle: SNPs common to all 3 genomes; Beijing K-family backbone SNPs; K-1, K-2 and H37Rv specific SNPs). Non-synonymous or inter-genic SNPs are shown as long lines whereas short lines represent synonymous SNPs. In two cases, where a SNP was non-synonymous in one gene and synonymous in a second, overlapping gene intermediate lines were used. The 5 resistance causing mutations in K-2 (Table 2) are highlighted separately in red on the outside. An equivalent, fully zoomable representation for each individual genome based on Artemis [25] is available in the Supplement (Files S3-S6).

Re-sequencing of H37Rv uncovered 80 SNPs with respect to the published reference genome. 74 SNPs were shared with the Beijing isolates sequenced in this study (Figure 2A), all of which were also detected in one or more studies of H37Ra, the avirulent form of H37 (File S1), by the following different experimental methodologies: a microarray-based comparative approach [26], re-sequencing [27] and a complete genome sequence (CP000611.1) from a shotgun library [28]. In contrast, the 6 H37Rv specific SNPs did not feature in any of these studies. Re-examination of the electropherograms of the original H37Rv sequence revealed that 44 of these SNPs were errors in the reference strain H37Rv data (File S1). The remaining differences might represent micro-evolutionary events that occurred while culturing of the different H37 variants in separate laboratories.

### Genomic diversity

Next, we compared K-1 and K-2 to H37Rv. Both Beijing isolates shared a common pool of 1,209 single-nucleotide-polymorphisms (SNPs) (Figure 2A) and five large scale deletions compared to H37Rv (Table 1). These Beijing-specific differences were congruent with a set of variants reported previously (File S1). An overview about all previously described SNPs is given in File S1. We found that the majority of the SNPs in K-1 and K-2 were non-synonymous (57% in both cases), which confirms previous findings [29] and is consistent with reduced purifying selection acting on Mtb [10] (Figure 2B).

**Table 1:** Large chromosomal deletions detected in both Beijing isolates K-1 and K-2 [57].

Name	Genes	Comment
RD105	*Rv0071-Rv0074*	robust Beijing marker
RD149	*Rv1572c-Rv1587c*	occurs in all Beijing isolates
RD152	*Rv1754c-Rv1762c*	occurs in all Beijing isolates
RD181	*Rv2262c-Rv2263*	variably deleted in Beijing isolates
RD207	*Rv2816c-Rv2820c*	occurs in all Beijing isolates

**Figure 3:**
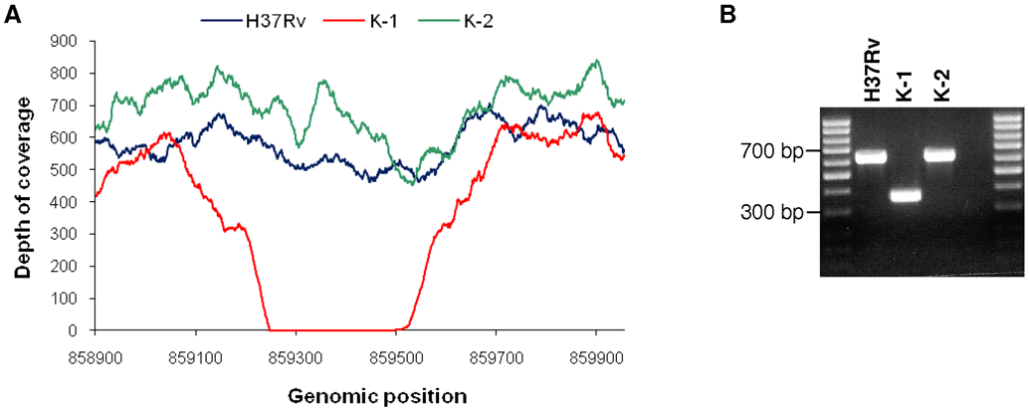
Genomic deletion specific for K-1. A, Graph showing the depth of coverage. A drop in coverage is visible in K-1 suggesting a deletion relative to the H37Rv reference. B, PCR and subsequent dideoxy sequencing (data not shown) identified a K-1 specific deletion at 859244-859501 (257 bp) affecting *cyp123* (*Rv0766c*) in correspondence with the coverage plot (Figure 3A).

In addition to this common backbone of Beijing specific variation, we identified 130 SNPs and one deletion that differentiated K-1 and K-2 (Figure 2A and Figure 3), although both isolates were identical with respect to IS*6110* typing (Figure 1). Compared to H37Rv, the susceptible isolate K-1 encoded 55 specific SNPs and one specific deletion, while the MDR variant K-2 had 75 specific SNPs, including the five resistance-conferring mutations in the *katG*, *rpoB*, *embB*, *pncA*, and *rpsL* genes that are known to be altered in resistance to INH, RIF, EMB, PZA, and SM, respectively [30] (Table 2, Figure 2C). Importantly, the observed level of diversity was independent of the MDR phenotype of K-2, as we detected comparable numbers of SNPs in both the drug-susceptible K-1 and the MDR K-2.

**Table 2:** SNPs in K-2 responsible for antibiotic resistances.

Antibiotic	Gene	Synonym	Position	Base change	Amino acid	Reference
Isoniazid	*katG*	*Rv1908c*	2155168	aGc/aCc	S315T	[48], [49] a
Rifampicin	*rpoB*	*Rv0667*	761161	cTg/cCg	L(s)452P^b^	[50]
Ethambutol	*embB*	*Rv3795*	4248003	cAg/cGg	Q497R	[51], [52]
Pyrazinamide	*pncA*	*Rv2043c*	2288885	tgG/tgA	W119*	[53], [54], [55] c
Streptomycin	*rpsL*	*Rv0682*	781687	aAg/aGg	K43R	[56]

a Mutation leads to high level resistance.

b Please note that this corresponds to amino acid 45**8** based on the TIGR annotation of H37Rv (http://cmr.jcvi.org/cgi-bin/CMR/GenomePage.cgi?org=ntmt02) in which the N-terminus was annotated to start 6 amino acids upstream of the start in TubercuList. The equivalent amino acid in *E. coli* is 533.

c For a detailed discussion of this mutation please refer to the Supplementary Results(File S1).

**Figure 4:**
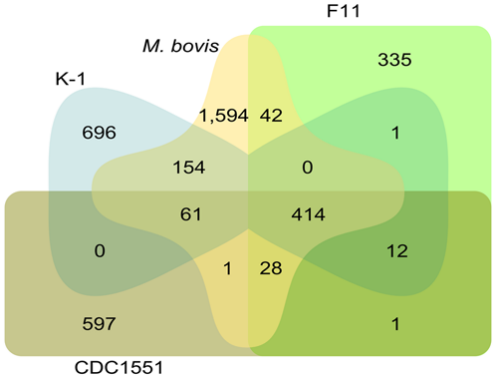
Comparison of SNPs across Mtb genomes. Edwards' Venn diagram showing the distribution of SNPs in K-1 (1,338 total) and the published genomes of CDC1551 (1,114 total), F11 (833 total) and *M. bovis* (2,294 total) relative to the published H37Rv sequence.

We then investigated the diversity between different lineages. Despite the fact that the total number of differences we observed between the Beijing isolates K-1/K-2 and the laboratory strain H37Rv was in the same range of the DNA variation described earlier between H37Rv and the clinical strain CDC1551 [29], a comparison to the whole genome sequences of CDC1551 (AE000516.2), F11 (CP000717.1) and *M. bovis* (BX248333.1) revealed that more than half of the SNPs in K-1 were in fact unique to the Beijing genotype (Figure 4).

## Discussion

Early sequencing studies of *M. tuberculosis* showed an extremely high level of clonality and conservation [31], [32]. As a consequence, standard molecular markers that are based on mobile or repetitive elements (IS*6110* RFLP, spoligotyping and MIRU-VNTR typing) are presumed to provide a high enough discriminatory power to accurately reconstruct transmission chains as part of epidemiological studies. Based on these standard criteria, our isolates K-1 and K-2 that exhibit the same molecular fingerprint can be considered as belonging to the same “outbreak clone” that is rapidly expanding in the study area by extensive transmission [22]. Therefore, we expected to find a low amount of genetic diversity.

By contrast, we observed a strikingly high genomic diversity among two isolates sharing the same DNA fingerprint. These findings challenge the “identical fingerprints – same strain” dogma and have important implications for the investigation of disease dynamics, especially in high incidence regions such as Eastern Europe where the Beijing genotype is dominant and the population diversity (e.g. among MDR strains) is significantly reduced [unpublished results,14,33]. The fact that strains with identical genotyping patterns can accumulate significant amounts of genetic diversity indicates that epidemiological links between isolates with identical genotyping data can be more remote and are likely to represent older transmission events rather than cases of recent transmission among patients in one RFLP cluster. Under these circumstances, a valid differentiation of relapse and re-infection, that can occur in significant numbers in TB patients undergoing standard treatment in areas with high rates of MDR [34], might not be possible by applying traditional genotyping.

One of the key objectives of molecular epidemiology in TB is to distinguish treatment failure/relapse with the same strain from exogenous reinfection with a second strain. This is particularly important given the emergence of MDR and extensively-drug resistant (XDR) TB in many parts of the world [4], [35], [36]. If a TB patient fails treatment, this indicates that the infecting strain might have become drug-resistant, or that the treatment is not effective, for example because of a lack of patient compliance. In the case of clinical trials where new TB drugs are being evaluated, treatment failure may suggest poor drug performance. Large amounts of resources are now being invested into the development of new drugs against TB [3]. Some of these new drug candidates are entering human trials. If during these trials treatment failures are not adequately differentiated from cases of exogenous re-infection, promising new drug candidates may be abandoned erroneously.

A large genetic diversity among closely related isolates of Mtb has also implications for our understanding of the evolution of drug resistance. A recent report detected only few differences when comparing the genomes of MDR and XDR Mtb strains from KwaZulu-Natal, South Africa to an isogenic drug-susceptible isolate (11 unique SNPs in the MDR and 15 in the DS and XDR, respectively) [37], [38]; the authors concluded that the evolution of drug resistance might be relatively easy to study [38]. We have previously shown that the fitness of drug-resistant Mtb depends both on the specific drug-resistance-conferring mutations and strain genetic backgrounds, and that compensatory mutations might mitigate the potential deleterious effects of drug resistance on bacterial fitness [8], [18]. Some of the strain specific mutations in K-2 might represent mutations which are compensating for putative fitness effects of the drug resistance-conferring mutations. However, more work is needed to explore this possibility.

Finally, the high number of novel SNPs that are unique to the Beijing lineage confirmed that previously sequenced genomes only sampled a small part of the global diversity of Mtb [9]. These findings highlight the importance of current sequencing efforts to capture the full global diversity of Mtb to ensure that both antibiotics and vaccines that are currently undergoing clinical trials [3] are efficacious against all strains.

In conclusion, our results showed that genomic diversity in closely related strains of Mtb may be greater than anticipated. Clonal variants can accumulate high levels of genomic diversity, which can lead to different pathogenic properties undetectable by standard genotyping [27]. Methods to accurately index strain diversity are essential for TB control, especially for the evaluation of new drugs, diagnostics and vaccines. Whole genome sequencing is likely to become available soon for routine molecular epidemiology at comparable costs to traditional typing techniques [39]. Instead of having to rely on molecular surrogates with a limited resolution, such an approach would be highly informative both by giving a more accurate picture of ongoing tuberculosis transmission and related epidemiological outcomes such as re-infection and mixed infections, as well as by shedding light on the microevolutionary processes occurring in *M. tuberculosis* during spread. In clinical settings, such an approach could allow for rapid detection of drug-resistant TB and enhanced disease surveillance.

## Methods

K-1 was isolated from sputum in 11/2001 and K-2 in 09/2004. IS*6110* RFLP fingerprints, spoligotype patterns and MIRU-VNTR profiles were determined as previously described [40], [41], [42], [43]. Antibiotic resistance was determined with the BACTEC MGIT 960 [44], [45].

∼200 bp paired read fragment libraries were prepared from 1 µg of DNA, and sequenced using the Illumina Genome Analyzer as described previously [23]. SNPs were mapped by paired short read alignment of individual reads to their cognate positions in the *M. tuberculosis* H37Rv genome (AL123456.2) and called at threshold ≥100 with the Illumina Genome Analyzer Pipeline. Where more than one base was called, only the majority call was accepted when the ratio between the two calls was ≥3. Only positions for which valid base calls were available in all genomes were considered for our assessment of the diversity. The validity of the SNP calls was confirmed by 40 kb of dideoxy chain terminator sequencing (ABI 3130xl) of each isolate. Deletions ≥250 bp were detected using combined paired read data and localised homology mapping across the deletion boundary.

Details of the annotation used for the SNP analysis and the graphical representation of the tabular results (File S2) with Artemis [25] (Files S3-6) can be found in the Supplementary Methods (File S1).

## Supporting Information

File S1 Description of supplementary information Supplementary Tables and Figures as well as Supplementary Methods and Results
(1.07 MB PDF)

File S2 Supplementary information - Excel file with all SNPs detected
(0.30 MB ZIP)

File S3 Contains the H37Rv genome (AL123456.2) and TubercuList R11 annotation, necessary for the graphical representation of all SNPs.
(1.96 MB ZIP)

File S4 contains the graphical representation of SNPs in H37Rv
(0.05 MB ZIP)

File S5 Contains the graphical representation of SNPs in K-1 (Section 2.2).
(0.05 MB ZIP)

File S6 Contains the graphical representation of SNPs in K-2 (Section 2.2).
(0.05 MB ZIP)
