# Supplementary material for: Genomic Diversity among Drug Sensitive and Multidrug Resistant Isolates of Mycobacterium tuberculosis with Identical DNA Fingerprints
Source: PLoS One. 2009 Oct 12;4(10):e7407. doi: 10.1371/journal.pone.0007407 (PMC2756628; doi:10.1371/journal.pone.0007407)
Supplement: File S1 — Description of supplementary information Supplementary Tables and Figures as well as Supplementary Methods and Results (1.07 MB PDF) [file pone.0007407.s001.pdf]

Supplementary Information

Summary

File S1 (PDF) contains all Supplementary Tables and Figures as well as Supplementary Methods and Results. `-- file currently opened --`

File S2 (zipped XLS) lists all SNPs detected (Section 2.1).

File S3 (zipped EMBL) contains the H37Rv genome (AL123456.2) and TubercuList R11 annotation, necessary for the graphical representation of all SNPs (Section 2.2).

File S4 (zipped TXT) contains the graphical representation of SNPs in H37Rv (Section 2.2).

File S5 (zipped TXT) contains the graphical representation of SNPs in K-1 (Section 2.2).

File S6 (zipped TXT) contains the graphical representation of SNPs in K-2 (Section 2.2).

Contents

1 Supplementary Tables and Figures **4**

2 Supplementary Methods **56**

2.1 SNPs 56

2.1.1 Position of change 56

2.1.2 Score 56

2.1.3 Base change 56

2.1.4 Triplet position 56

2.1.5 (Amino acid) Change 57

2.1.6 Comment I 57

2.1.7 Comment II 57

2.1.8 Operon 1-5 58

2.1.9 Nature of genes affected 58

2.1.10 SNPs across genomes 59

2.2 Graphical display of results 60

2.2.1 Opening the files 60

2.2.2 The output 62

3 Supplementary Results **63**

3.1 Drug resistance 63

List of Supplementary Tables

1 Overview of all SNPs in H37Rv 5
2 Filliol global phylogeny: Table 3 9
3 Filliol global phylogeny: Table 4 11
4 Alland global phylogeny 12
5 Previously described synonymous SNPs 13
6 Previously described non-synonymous SNPs 32
7 Previously described inter-genic SNPs 48

List of Supplementary Figures

1 Graphical representation of SNPs in K-1. 4

1 Supplementary Tables and Figures

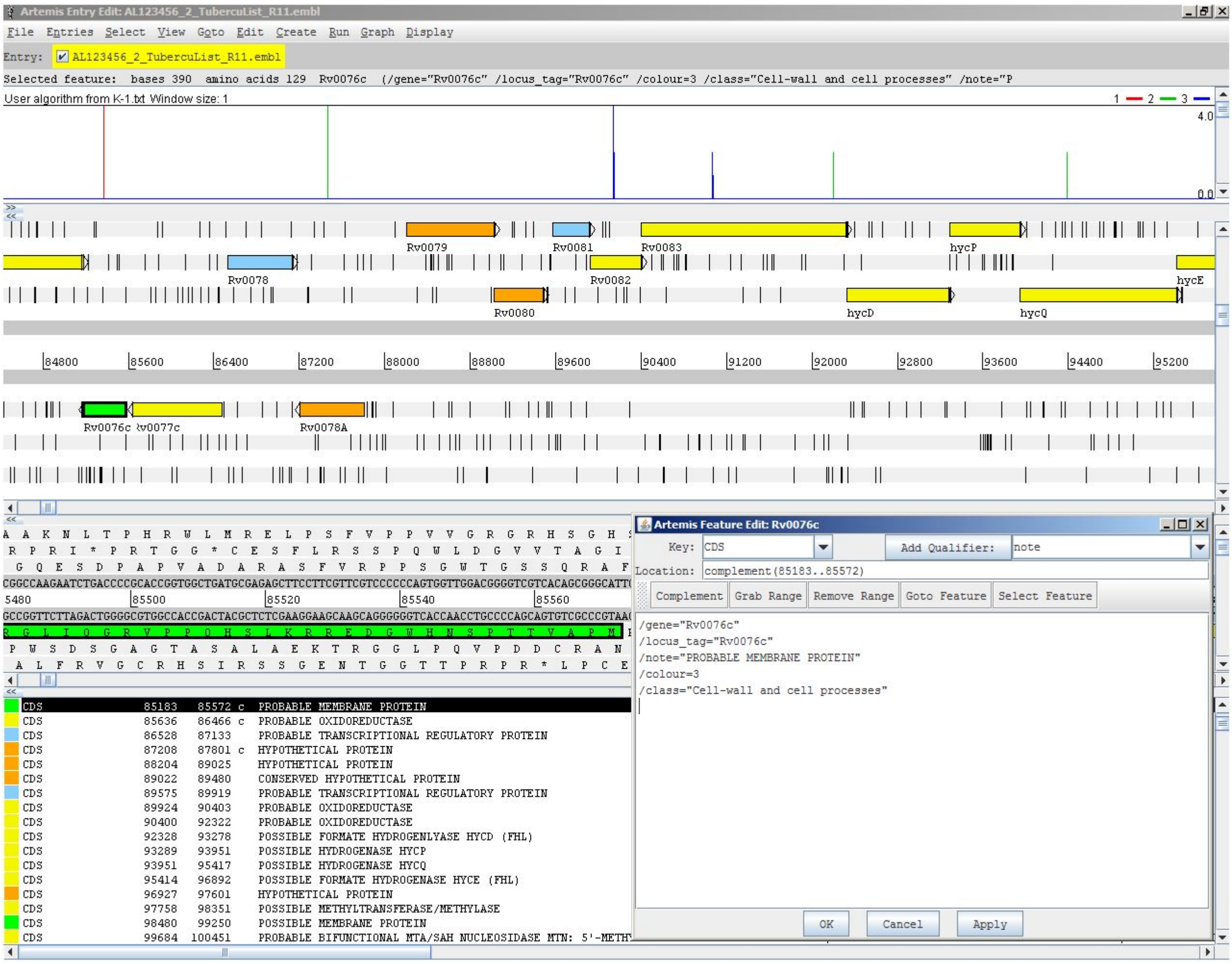

Supplementary Figure 1: **Graphical representation of SNPs in K-1.**
The summary information for *Rv0076c* is visible in the bottom right corner. Also, 6 SNPs are within the field of view:

a) One non-synonymous SNP specific to K-1 in *Rv0076c*.
b) One non-synonymous Beijing K-family backbone SNP in *Rv0078A*.
c) Two SNPs common to all 3 genomes (non-synonymous in *Rv0082* and synonymous in *Rv0083*).
d) Two synonymous Beijing K-family backbone SNPs in *Rv0083* and *hycQ*.

Please refer to Section 2.2 for more details.

Supplementary Table 1: **Overview of the 80 SNPs observed in H37Rv** and comparison to previous studies of H37Ra (Frigui *et al.* 2008 (F), Lee *et al.* 2008[a] (L) and Zheng *et al.* 2008 (Z)). Errors in the reference strain H37Rv data are highlighted in blue[b].

Gene	Synonym	Position	Base change	Amino acid	F	L	Z
Rv0012		14785	Tgc/Cgc	C233R		x	x
Rv0064		69989	gGt/gAt	G457D	x	x	x
Rv0082		90144	cAa/cGa	Q74R		x	x
Rv0083		91071	atT/atC	I(s)224I(s)		x	x
nrp	*Rv0101*	116000	gtT/gtG	V2000V(s)	x	x	x
PE_PGRS1	*Rv0109*	132417	Cgc/Ggc	R346G		x	x
Rv0204c		242299	Gtg/Ctg	V(s)306L(s)		x	x
Rv0323c		390828	Agt/Ggt	S142G	x		x
PPE8	*Rv0355c*	426909	Tgg/Ggg	W2591G		x	x
pyrE	*umpA, Rv0382c*	458282	taT/taC	Y33Y		x	x
Rv0383c-clpB		459399	A/C	84, 57[c]		x	
PPE9	*Rv0388c*	467526	gGg/gCg	G159A			x
		467546	gaC/gaG	D152E			x
		467557	Ttg/Gtg	L(s)149V(s)			x
		467564	caT/caG	H146Q			x
		467585	caC/caG	H139Q			x
		467590	Acg/Gcg	T138A			x
		467621	ggA/ggC	G127G		x	x
		467638	Cag/Aag	Q122K		x	x
ctpH	*Rv0425c*	511518	atA/atC	I(s)1268I(s)		x	x
PPE10	*Rv0442c*	532097	Aaa/Gaa	K40E		x	x
Rv0461		552085	caA/caG	Q20Q		x	x
Rv0473		563577	aAa/aGa	K5R		x	x
PE_PGRS6	Rv0532	623508	gCc/gGc	A239G		x	x
Rv0543c		635633[d]	gcG/gcA	A81A			
PE_PGRS7	*Rv0578c*	672491	ggG/ggC	G1142G		x	x

Supplementary Table 1: (continued)

Gene	Synonym	Position	Base change	Amino acid	F	L	Z
rpsL	*Rv0682*	781922	aaA/aaG	K121K		x	x
PE_PGRS9	*Rv0746*	836658	Acc/Gcc	T320A			x
		837033	Acc/Gcc	T445A			x
ercc3	*Rv0861c*	958922[d]	gcG/gcT	A410A			
Rv0890c		990001	Ccc/Gcc	P866A		x	x
Rv0919		1025106	ttT/ttC	F141F	x	x	x
pstA1	*Rv0930*	1037911	Cga/Tga	R305*		x	x
PE_PGRS17	*Rv0978c*	1093406	gtT/gtC	V317V		x	x
zwf1	*Rv1121, zwf*	1244700	Tta/Cta	L332L		x	x
pks3	*Rv1180*	1315191	taA/taC	*489Y	x	x	x
pks4	*Rv1181*	1315884	gcG/gcA	A217A	x	x	x
fadD21	*Rv1185c*	1327402	gaA/gaG	E37E		x	x
Rv1188		1331696	Agg/Cgg	R226R		x	x
pknH	*Rv1266c*	1414021	cGg/cAg	R607Q	x	x	x
rho	*Rv1297*	1453608	ggT/ggC	G135G		x	x
murA-rrs		1471659	C/T	82, 187[c]		x	x
Rv1520		1711627[d]	taC/taT	Y200Y			
dsbF	*Rv1677*	1901816	caA/caG	Q23Q		x	x
Rv1771		2006032[d]	cAa/cGa	Q291R			
Rv1783		2020563	tAg/tTg	*436L(s)		x	x
PPE32	*Rv1808*	2050913	gaA/gaG	E331E		x	x
PPE33	*Rv1809*	2051746	gcT/gcC	A155A	x	x	x
Rv1815		2057774	Atc/Ttc	I(s)83F	x	x	x
Rv1907c		2153410[d]	gTt/gCt	V158A			
PPE34-PPE35		2167489	T/C	178, 160[c]			x
fadD31	Rv1925	2177654	Atg/Ctg	M(s)190L(s)			x

Supplementary Table 1: (continued)

Gene	Synonym	Position	Base change	Amino acid	F	L	Z
Rv1977		2219929[d]	cTg/cCg	L(s)59P			
Rv1979c		2221796	Gtc/Atc	V457I(s)		x	x
Rv2005c-otsB1		2251999	A/G	116, 3[c]		x	x
Rv2037c		2282787	tGt/tAt	C312Y	x	x	x
pks12	*Rv2048c*	2297976	tCa/tTa	S3004L		x	x
helZ	*Rv2101*	2361623	Atg/Ctg	M(s)462L(s)		x	x
PE_PGRS37	*Rv2126c*	2387733	gaA/gaG	E80E		x	x
Rv2205c		2470149	gaA/gaG	E105E		x	x
Rv2231A		2505919	gcT/gcC	A81A		x	x
nadD-Rv2422		2718852	T/G	44, 231[c]		x	x
rpfE	*Rv2450c*	2751804	cGg/cAg	R126Q		x	x
pdhC	*Rv2495c*	2809621	Acc/Gcc	T107A	x	x	x
Rv2614A		2943411	ctT/ctC	L12L		x	x
Rv2627c		2954439	Aga/Gga	R104G		x	x
Rv2680		2996194	gtT/gtA	V30V		x	x
Rv2695		3012293	acA/acG	T126T		x	x
Rv2896c		3205978	Tcg/Gcg	S153A		x	x
ppsB	*Rv2932*	3254365	ctT/ctC	L1098L		x	x
gatA	*Rv3011c*	3370177	Atg/Ctg	M(s)420L(s)		x	x
PPE47	*Rv3021c*	3379708	Ctg/Gtg	L(s)249V(s)			x
		3379712	gcC/gcG	A247A			x
		3379784	ggG/ggT	G223G			x
		3379788	gGg/gCg	G222A			x
PPE52	*Rv3144c*	3510642	Agc/Ggc	S226G		x	x
sugI	*Rv3331*	3718357	cCg/cTg	P423L(s)			x
Rv3479		3896340	cTc/cGc	L174R		x	x

Supplementary Table 1: (continued)

Gene	Synonym	Position	Base change	Amino acid	F	L	Z
gshA	*Rv3704c*	4147070	Ttg/Ctg	L(s)373L(s)		x	x
gid	*gidB, Rv3919c*	4407904	tCt/tTt	S100F			x

[a]Our thanks to Roland Krause from the Department of Computational Molecular Biology (Max Planck Institute for Molecular Genetics) for translating the H37Ra positions into the corresponding one in H37Rv.

[b]Our thanks to Julian Parkhill (Wellcome Trust Sanger Institute) for this re-examination.

[c]Distance in base pairs to the two genes located nearest on either side (see Section 2.1.5).

[d]This SNP is specific to H37Rv.

Supplementary Table 2: Translation of positions of the **global phylogeny (Filliol *et al.* 2006: Table 3)** into the corresponding positions of the current H37Rv genome[a]. Unless otherwise stated, the bases determined in this study were the same as in the published reference H37Rv sequence. "–" denotes the absence of sequence information that meets the criteria detailed in the Methods. Accordingly, H37Rv was part of SNP cluster group 6b (sequence tag 40) and both Beijing isolates, K-1 and K-2, of group 2 (sequence tag 10)[b].

Position in Filliol	Difference	Current position[c]	Reference	H37Rv	K-1	K-2
37031	0	37031	C		G	G
43943	2	43945	A			
92197	2	92199	T		G	G
220048	2	220050	C			
311611	2	311613	G		T	T
519804	2	519806	G			
797736	0	797736	C		T	T
909164	2	909166	C	-	-	-
918314	2	918316	T			
923063	2	923065	T			
949219	2	949221	C			
1068149	2	1068151	T		C	C
1163132	2	1163134	T		C	C
1191859	2	1191861	T			
1294396	2	1294398	C			
1477596	0	1477596	C		T	T
1548147	2	1548149	G			
1692067	2	1692069	A			
1692683	2	1692685	C			
1884695	2	1884697	G			
1892015	2	1892017	T		C	C
1952599	2	1952601	C			
2158580	2	2158582	G			
2223680	2	2223682	G			
2376133	2	2376135	A			
2462869	2	2462871	G		A	A
2532614	2	2532616	G			
2627946	0	2627946	A		G	G
2825579	2	2825581	T		G	G
2891265	2	2891267	C		T	T
2990037	3	2990040	C			
3207247	3	3207250	A			
3438383	3	3438386	G			
3440461	3	3440464	T		G	G
3440539	3	3440542	A			
3450722	3	3450725	T		C	C

Supplementary Table 2: (continued)

Position in Filliol	Difference	Current position[c]	Reference	H37Rv	K-1	K-2
3455683	3	3455686	G		C	C
3544707	3	3544710	T			
3783054	4	3783058	G			
4024270	3	4024273	T		C	C
4119243	3	4119246	T			
4137829	0	4137829	C		T	T
4254003	3	4254006	G	–	–	–
4255919	3	4255922	A		G	G
4280708	0	4280708	G		A	A

[a]Please refer to Supplementary Tables 5-7 for a detailed description of the listed SNPs.

[b]Our thanks to Alifiya S. Motiwala from the University of Medicine and Dentistry of New Jersey for translating the positions.

[c]AL123456.2

Supplementary Table 3: Translation of positions of the **global phylogeny (Filliol *et al.* 2006: Table 4)** into the corresponding positions of the current H37Rv genome[a]. Unless otherwise stated, the bases determined in this study were the same as in the published reference H37Rv sequence. Accordingly, H37Rv was part of SNP cluster group 6 and both Beijing isolates, K-1 and K-2, of group 2[b].

Position in Filliol	Difference	Current position[c]	Reference	H37Rv	K-1	K-2
92197[d]	2	92199	T		G	G
473687	2	473689	G			
519804	2	519806	G			
1830293	2	1830295	G			
1920118	2	1920120	G			
2156023	2	2156025	G			
2361602	2	2361604	C		G	G
2376133	2	2376135	A			
2460626	2	2460628	C			
2598398	2	2598400	A		G	G
3057134	3	3057137	C			
3111473	3	3111476	C		T	T
3352929	3	3352932	C		G	G
3440539	3	3440542	A			
3550786	3	3550789	C		G	G
4314642	3	4314645	A		G	G

[a]Please refer to Supplementary Tables 5-7 for a detailed description of the listed SNPs.

[b]Our thanks to Alifiya S. Motiwala from the University of Medicine and Dentistry of New Jersey for translating the positions.

[c]AL123456.2

[d]Please note that this is the correct position. 192197 in the paper is a typographical error (Alifiya S. Motiwala, personal communication).

Supplementary Table 4: Positions of the **global phylogeny (Alland *et al.* 2007)**[a]. Unless otherwise stated, the bases determined in this study were the same as in the published reference H37Rv sequence. Accordingly, H37Rv was part of SNP cluster group 6b and both Beijing isolates, K-1 and K-2, of group 2.

Position	Reference	H37Rv	K-1	K-2
1977	A		G	G
54394	A		G	G
74092	C			
105139	C		A	A
144390	G			
232574	G			
311613	G		T	T
913274	C			
2154724	C		A	A

[a]Please refer to Supplementary Tables 5-7 for a detailed description of the listed SNPs.

Supplementary Table 5: **Previously described synonymous SNPs.** Unless otherwise stated in *italic font*, they were part of the Beijing K-family backbone[a] of both isolates. Comments were taken from the publications in question.

Gene	Synonym	Position	Base change	Amino acid	Reference & comment
Rv0034		37031	gcC/gcG	A55A	Gutacker *et al.* 2002; Filliol *et al.* 2006: global phylogeny *(Supplementary Table 2)*; Liu *et al.* 2006;
mtc28	*Rv0040c*	42967	ccC/ccG	P133P	Liu *et al.* 2006; Hershberg *et al.* 2008
Rv0044c		48503	cgA/cgG	R175R	Gutacker *et al.* 2002; Filliol *et al.* 2006
ponA1	Rv0050	54394	gcA/gcG	A244A	Gutacker *et al.* 2002; Filliol *et al.* 2006; Liu *et al.* 2006; Alland *et al.* 2007: global phylogeny *(Supplementary Table 4)*
Rv0083		91071	atT/atC	I(s)224I(s)	Lee *et al.* 2008[b]; Zheng *et al.* 2008; *common to all 3 genomes, error in original H37Rv sequence (Supplementary Table 1)*
		92199	acT/acG	T600T	Gutacker *et al.* 2002; Filliol *et al.* 2006: global phylogeny *(Supplementary Tables 2 and 3)*; Gutacker *et al.* 2006: part of 36 SNP-set; Liu *et al.* 2006: part of 36 SNP-set in data set I
Rv0095c		105011	Ctg/Ttg	L(s)69L(s)	Liu *et al.* 2006: inter-genic at distance 438 in IRMT0105 in data set II
		105021	tcC/tcT	S65S	Liu *et al.* 2006:

Supplementary Table 5: (continued)

Gene	Synonym	Position	Base change	Amino acid	Reference & comment
					inter-genic at distance 448 in IRMT0105 in data set II
nrp	*Rv0101*	116000	gtT/gtG	V2000V(s)	Frigui *et al.* 2008; Lee *et al.* 2008[b]; Zheng *et al.* 2008; *common to all 3 genomes (Supplementary Table 1)*
PE1	*Rv0151c*	177857	Ctg/Ttg	L(s)485L(s)	Liu *et al.* 2006
Rv0165c		194681	ctC/ctG	L86L(s)	Gutacker *et al.* 2002; Filliol *et al.* 2006; Liu *et al.* 2006
fadD5	Rv0166	196642	aaC/aaT	N550N	Filliol *et al.* 2006
bglS	*Rv0186*	217201	aaT/aaC	N311N	Gutacker *et al.* 2002
Rv0195		231114	gcC/gcG	A72A	Gutacker *et al.* 2006: part of 36 SNP-set; Liu *et al.* 2006: part of 36 SNP-set in data set I
Rv0260c		311613	gtC/gtA	V349V	Gutacker *et al.* 2002; Filliol *et al.* 2006: global phylogeny *(Supplementary Table 2)*; Liu *et al.* 2006; Alland *et al.* 2007: global phylogeny *(Supplementary Table 4)*
fadD2	*Rv0270*	325505	gtT/gtC	V313V	Gutacker *et al.* 2002; Liu *et al.* 2006
Rv0307c		376774	gcA/gcG	A94A	Gutacker *et al.* 2002; Gutacker *et al.* 2006: part of 36 SNP-set; Liu *et al.* 2006: part of 36 SNP-set in data set I

Supplementary Table 5: (continued)

Gene	Synonym	Position	Base change	Amino acid	Reference & comment
lpqJ	*Rv0344c*	414486	gaG/gaA	E152E	Liu *et al.* 2006
dnaK	*hsp70*, *Rv0350*	420008	gcA/gcG	A58A	Gutacker *et al.* 2002; Filliol *et al.* 2006; Liu *et al.* 2006
pyrE	*umpA*, *Rv0382c*	458282	taT/taC	Y33Y	Lee *et al.* 2008[b]; Zheng *et al.* 2008; *common to all 3 genomes, error in original H37Rv sequence (Supplementary Table 1)*
PPE9	*Rv0388c*	467621	ggA/ggC	G127G	Lee *et al.* 2008[b]; Zheng *et al.* 2008; *common to all 3 genomes, error in original H37Rv sequence (Supplementary Table 1)*
fgd1	*fgd*, *Rv0407*	491742	ttT/ttC	F320F	Gutacker *et al.* 2002; Hershberg *et al.* 2008
Rv0412c		498531	gcT/gcC	A363A	Gutacker *et al.* 2002
ctpH	*Rv0425c*	511518	atA/atC	I(s)1268I(s)	Lee *et al.* 2008[b]; Zheng *et al.* 2008; *common to all 3 genomes, error in original H37Rv sequence (Supplementary Table 1)*
mmpL4	*Rv0450c*	541201	ctT/ctC	L97L	Gutacker *et al.* 2002; Filliol *et al.* 2006; Liu *et al.* 2006
Rv0459		551525	Aga/Cga	R110R	Gutacker *et al.* 2002; Liu *et al.* 2006
Rv0461		552085	caA/caG	Q20Q	Lee *et al.* 2008[b]; Zheng *et al.* 2008; *common to all 3 genomes, error in original H37Rv sequence (Supplementary*

Supplementary Table 5: (continued)

Gene	Synonym	Position	Base change	Amino acid	Reference & comment
					Table 1)
proC	*Rv0500*	590436	gcT/gcC	A118A	Gutacker *et al.* 2002; Liu *et al.* 2006
Rv0575c		669398	caA/caG	Q116Q	Gutacker *et al.* 2002; Liu *et al.* 2006
PE_PGRS7	*Rv0578c*	672491	ggG/ggC	G1142G	Lee *et al.* (2008)[b]; Zheng *et al.* 2008; *common to all 3 genomes (Supplementary Table 1)*
Rv0585c		683829	gcG/gcA	A148A	Gutacker *et al.* 2002; Gutacker *et al.* 2006: part of 36 SNP-set; Liu *et al.* 2006: part of 36 SNP-set in data set I
Rv0601c		698968	ggC/ggT	G9G	Deshayes *et al.* 2008
recC	*Rv0631c*	725190	ttC/ttT	F1039F	Dos Vultos *et al.* 2008
rpoB	*Rv0667*	763031	gcT/gcC	A1075A[c]	Gutacker *et al.* 2002; Baker *et al.* 2004: T marker for lineage II; Liu *et al.* 2006; Gagneux *et al.* 2007: T marker for lineage II
rpsL	*Rv0682*	781922	aaA/aaG	K121K	Siddiqi *et al.* 2002; Sekiguchi *et al.* 2007: present in all isolates tested irrespective of resistance status; Lee *et al.* 2008[b]; Zheng *et al.* 2008; *common to all 3 genomes, error in original H37Rv sequence (Supplementary Table 1)*
Rv0697		797736	ctC/ctT	L268L	Filliol *et al.* 2006: global phylogeny *(Supple-*

Supplementary Table 5: (continued)

Gene	Synonym	Position	Base change	Amino acid	Reference & comment
					mentary Table 2), Beijing specific; Iwamoto *et al.* 2008
Rv0764		857696	gcT/gcC	A114A	Gutacker *et al.* 2002; Gutacker *et al.* 2006: part of 36 SNP-set; Liu *et al.* 2006: part of 36 SNP-set in data set I
cpsY	*Rv0806c*	900221	gtA/gtG	V370V(s)	Gutacker *et al.* 2002; Filliol *et al.* 2006; Liu *et al.* 2006
purF	*Rv0808*	903550	gcT/gcC	A480A	Gutacker *et al.* 2002; Liu *et al.* 2006
purM	*Rv0809*	903913	ggT/ggC	G63G	Gutacker *et al.* 2002; Filliol *et al.* 2006; Liu *et al.* 2006
lpqQ	*Rv0835*	931123	taT/taC	Y57Y	Gutacker *et al.* 2002; Liu *et al.* 2006
pdc	*Rv0853c*	949535	gcA/gcG	A528A	Gutacker *et al.* 2002; Liu *et al.* 2006
fadB	*Rv0860*	957117	gaT/gaC	D275D	Gutacker *et al.* 2002; Gutacker *et al.* 2006; Filliol *et al.* 2006; Liu *et al.* 2006; Deshayes *et al.* 2008
Rv0919		1025106	ttT/ttC	F141F	Frigui *et al.* 2008; Lee *et al.* 2008[b]; Zheng *et al.* 2008; *common to all 3 genomes (Supplementary Table 1)*
pstS2	*Rv0932c*	1040251	ccG/ccT	P266P	Filliol *et al.* 2006
purN	*Rv0956*	1068151	caT/caC	H197H	Gutacker *et al.* 2002; Filliol *et al.* 2006: global phylogeny *(Supple-*

Supplementary Table 5: (continued)

Gene	Synonym	Position	Base change	Amino acid	Reference & comment
					mentary Table 2); Gutacker *et al.* 2006: part of 36 SNP-set; Liu *et al.* 2006: part of 36 SNP-set in data set I
purH	*Rv0957*	1068432	ccA/ccG	P76P	Gutacker *et al.* 2002; Filliol *et al.* 2006; Liu *et al.* 2006
ctpV	*Rv0969*	1079927	acC/acA	T395T	Gutacker *et al.* 2002; Liu *et al.* 2006
PE_PGRS17	*Rv0978c*	1093406	gtT/gtC	V317V	Lee *et al.* 2008[b]; Zheng *et al.* 2008; *common to all 3 genomes, error in original H37Rv sequence (Supplementary Table 1)*
kdpD	*Rv1028c*	1149551	gaG/gaA	E712E	Gutacker *et al.* 2002; Filliol *et al.* 2006; Liu *et al.* 2006
PE8	*Rv1040c*	1163134	ggA/ggG	G81G	Gutacker *et al.* 2002; Filliol *et al.* 2006: global phylogeny *(Supplementary Table 2)*; Liu *et al.* 2006
Rv1056		1178116	acT/acC	T163T	Gutacker *et al.* 2002; Filliol *et al.* 2006; Liu *et al.* 2006
zwf1	*Rv1121*, *zwf*	1244700	Tta/Cta	L332L	Lee *et al.* 2008[b]; Zheng *et al.* 2008; *common to all 3 genomes, error in original H37Rv sequence (Supplementary Table 1)*
Rv1125		1248936	ccG/ccC	P285P	Gutacker *et al.* 2006

Supplementary Table 5: (continued)

Gene	Synonym	Position	Base change	Amino acid	Reference & comment
		1248978	gcT/gcC	A299A	Gutacker *et al.* 2002
narH	*Rv1162*	1292102	ccA/ccG	P346P	Gutacker *et al.* 2002; Filliol *et al.* 2006; Liu *et al.* 2006
pks4	*Rv1181*	1315884	gcG/gcA	A217A	Frigui *et al.* 2008; Lee *et al.* 2008[b]; Zheng *et al.* 2008; *common to all 3 genomes (Supplementary Table 1)*
fadH	*Rv1175c*	1306259	gcT/gcC	A656A	Gutacker *et al.* 2002; Gutacker *et al.* 2006: part of 36 SNP-set; Liu *et al.* 2006: part of 36 SNP-set in data set I
fadD21	*Rv1185c*	1327402	gaA/gaG	E37E	Lee *et al.* 2008[b]; Zheng *et al.* 2008; *common to all 3 genomes, error in original H37Rv sequence (Supplementary Table 1)*
Rv1186c		1327890	gaC/gaT	D472D	Liu *et al.* 2006;
		1329234	gaC/gaT	D24D	Filliol *et al.* 2006
Rv1188		1331696	Agg/Cgg	R226R	Lee *et al.* 2008[b]; Zheng *et al.* 2008; *common to all 3 genomes, error in original H37Rv sequence (Supplementary Table 1)*
Rv1249c		1393626	ctT/ctC	L119L	Gutacker *et al.* 2002; Filliol *et al.* 2006; Gutacker *et al.* 2006: part of 36 SNP-set; Liu *et al.* 2006: part of 36 SNP-set in data set I

Supplementary Table 5: (continued)

Gene	Synonym	Position	Base change	Amino acid	Reference & comment
Rv1251c		1396922	acA/acG	T773T	Gutacker *et al.* 2002; Liu *et al.* 2006
amiB2	*Rv1263*	1411210	gtT/gtG	V260V(s)	Liu *et al.* 2006
cysN	*Rv1286*	1440469	ccC/ccG	P521P	Liu *et al.* 2006
rho	*Rv1297*	1453608	ggT/ggC	G135G	Lee *et al.* 2008[b]; Zheng *et al.* 2008; *common to all 3 genomes, error in original H37Rv sequence (Supplementary Table 1)*
ogt	*Rv1316c*	1477596	ggG/ggA	G12G	Rad *et al.* 2003: Beijing specific; Filliol *et al.* 2006: global phylogeny *(Supplementary Table 2)*, found in SCG 2 and 3b; Olano *et al.* 2007: Beijing specific; Dos Vultos *et al.* 2008; Hershberg *et al.* 2008; Iwamoto *et al.* 2008
Rv1358		1526819	Cgg/Agg	R70R	Gutacker *et al.* 2002; Filliol *et al.* 2006; Liu *et al.* 2006
ribC	*Rv1412*	1588899	gcG/gcT	A111A	Gutacker *et al.* 2002; Filliol *et al.* 2006; Liu *et al.* 2006
PE16	*Rv1430*	1606673	gcG/gcT	A96A	Gutacker *et al.* 2002; Filliol *et al.* 2006
Rv1501		1692141	atA/atC	I84I	Gutacker *et al.* 2002; Filliol *et al.* 2006; Gutacker *et al.* 2006: part of 36 SNP-set; Liu *et al.* (2006): part of 36 SNP-set in data set I

Supplementary Table 5: (continued)

Gene	Synonym	Position	Base change	Amino acid	Reference & comment
Rv1508c		1698911	ggC/ggT	G328G	Gutacker *et al.* 2002; Liu *et al.* 2006
fadD25	*Rv1521*	1713192	gtA/gtG	V297V(s)	Gutacker *et al.* 2002; Filliol *et al.* 2006; Liu *et al.* 2006
frdA	*Rv1552*	1759252	tcG/tcT	S524S	Gutacker *et al.* 2002; Filliol *et al.* 2006; Gutacker *et al.* 2006: part of 36 SNP-set; Liu *et al.* 2006: part of 36 SNP-set in data set I
Rv1514c		1706119	tcA/tcG	S159S	Liu *et al.* 2006
fadD25	*Rv1521*	1713192	gtA/gtG	V297V(s)	Liu *et al.* 2006
frdA	*Rv1552*	1759252	tcG/tcT	S524S	Liu *et al.* 2006
Rv1592c		1792778	gaA/gaG	E321E	Ramaswamy *et al.* 2003: observed in all group 1, some group 2 but not in any group 3 isolates
polA	*Rv1629*	1831288	ccC/ccT	P208P	Dos Vultos *et al.* 2008
rpsA	*Rv1630*	1834177	cgA/cgC	R212R	Gutacker *et al.* 2002; Filliol *et al.* 2006
Rv1639c		1847919	acG/acC	T180T	Gutacker *et al.* 2002; Liu *et al.* 2006
pks11	*Rv1665*	1892017	caT/caC	H264H	Gutacker *et al.* 2002; Filliol *et al.* 2006: global phylogeny *(Supplementary Table 2)*, Beijing specific; Gutacker *et al.* 2006: part of 36 SNP-set; Liu *et al.* 2006: part of 36 SNP-set in data set I;

Supplementary Table 5: (continued)

Gene	Synonym	Position	Base change	Amino acid	Reference & comment
					Iwamoto *et al.* 2008
Rv1672c		1897608	ttG/ttA	L(s)200L	Gutacker *et al.* 2002
Rv1676		1901493	agT/agC	S149S	Gutacker *et al.* 2002; Filliol *et al.* 2006; Liu *et al.* 2006
dsbF	*Rv1677*	1901816	caA/caG	Q23Q	Lee *et al.* 2008[b]; Zheng *et al.* 2008; *common to all 3 genomes, error in original H37Rv sequence (Supplementary Table 1)*
Rv1691		1916137	ctA/ctG	L63L(s)	Filliol *et al.* 2006
Rv1724c		1950767	aaA/aaG	K95K	Filliol *et al.* 2006
Rv1735c		1961735	gcT/gcC	A18A	Gutacker *et al.* 2002; Filliol *et al.* 2006; Gutacker *et al.* 2006: part of 36 SNP-set; Liu *et al.* 2006: part of 36 SNP-set in data set I
Rv1784		2022868	agT/agC	S745S	Gutacker *et al.* 2002; Liu *et al.* 2006
esxM	*TB11.0*, *QILSS*, *Rv1792*	2030355	tcA/tcG	S3S	Deshayes *et al.* 2008; *specific to K-2*
PPE32	*Rv1808*	2050913	gaA/gaG	E331E	Lee *et al.* 2008[b]; Zheng *et al.* 2008; *common to all 3 genomes, error in original H37Rv sequence (Supplementary Table 1)*
PPE33	*Rv1809*	2051746	gcT/gcC	A155A	Frigui *et al.* 2008; Lee *et al.* 2008[b]; Zheng *et al.* 2008; *common to all 3 genomes*

Supplementary Table 5: (continued)

Gene	Synonym	Position	Base change	Amino acid	Reference & comment
					(Supplementary Table 1)
Rv1865c		2112832	gcT/gcG	A45A	Gutacker *et al.* 2002; Filliol *et al.* 2006
glnA3	*Rv1878*	2128870	ctA/ctG	L283L(s)	Gutacker *et al.* 2002; Filliol *et al.* 2006; Gutacker *et al.* 2006: part of 36 SNP-set; Liu *et al.* 2006: part of 36 SNP-set in data set I
fbpB	*85B*, *mpt59*, *Rv1886c*	2135154	ccC/ccA	P238P	Musser *et al.* 2000; Hershberg *et al.* 2008
mce3C	*Rv1968*	2211826	aaA/aaG	K67K	Gutacker *et al.* 2002; Liu *et al.* 2006
Rv1977		2220512	tcT/tcG	S253Z	Gutacker *et al.* 2002; Liu *et al.* 2006
Rv1985c		2229801	ccG/ccC	P34P	Gutacker *et al.* 2002; Liu *et al.* 2006
Rv2041c		2287121	gaT/gaC	D242D	Gutacker *et al.* 2002; Filliol *et al.* 2006; Liu *et al.* 2006
Rv2077c		2334007	gcT/gcC	A96A	Gutacker *et al.* 2002; Filliol *et al.* 2006; Liu *et al.* 2006
pknJ	*Rv2088*	2345037	ctC/ctA	L209L	Gutacker *et al.* 2002; Filliol *et al.* 2006; Liu *et al.* 2006
helZ	*Rv2101*	2361604	gtC/gtG	V455V(s)	Gutacker *et al.* 2002; Filliol *et al.* 2006: global phylogeny *(Supplementary Table 3)*; Liu *et al.* 2006
PE_PGRS37	*Rv2126c*	2387733	gaA/gaG	E80E	Lee *et al.* 2008[b]; Zheng *et al.* 2008;

Supplementary Table 5: (continued)

Gene	Synonym	Position	Base change	Amino acid	Reference & comment
					common to all 3 genomes (Supplementary Table 1)
mmpS3	*Rv2198c*	2462871	gcC/gcT	A59A	Gutacker *et al.* 2002; Filliol *et al.* 2006: global phylogeny *(Supplementary Table 2)*; Liu *et al.* 2006
Rv2205c		2470149	gaA/gaG	E105E	Gutacker *et al.* 2002; Lee *et al.* 2008[b]; Zheng *et al.* 2008; *common to all 3 genomes, error in original H37Rv sequence (Supplementary Table 1)*
Rv2231A		2505919	gcT/gcC	A81A	Lee *et al.* 2008[b]; Zheng *et al.* 2008; *common to all 3 genomes, error in original H37Rv sequence (Supplementary Table 1)*
Rv2237		2509722	ccA/ccG	P78P	Gutacker *et al.* 2002; Filliol *et al.* 2006
accD6	*Rv2247*	2521342	gaT/gaC	D200D	Ramaswamy *et al.* 2003; Liu *et al.* 2006
Rv2256c		2529680	acT/acC	T65T	Gutacker *et al.* 2002; Filliol *et al.* 2006; Liu *et al.* 2006
Rv2251		2526974	ccT/ccC	P470P	Deshayes *et al.* 2008
Rv2258c		2531742	gcT/gcC	A52A	Gutacker *et al.* 2002; Filliol *et al.* 2006; Gutacker *et al.* 2006: part of 36 SNP-set; Liu *et al.* 2006: part of 36 SNP-set in data set I

Supplementary Table 5: (continued)

Gene	Synonym	Position	Base change	Amino acid	Reference & comment
cyp128	*Rv2268c*	2543395	gaA/gaG	E294E	Gutacker *et al.* 2002
Rv2326c		2598400	aaT/aaC	N516N	Gutacker *et al.* 2002; Filliol *et al.* 2006: global phylogeny *(Supplementary Table 3)*; Liu *et al.* 2006
Rv2337c		2612256	ctC/ctA	L244L	Gutacker *et al.* 2002
Rv2426c		2723506	ttA/ttG	L226L(s)	Gutacker *et al.* 2002
plcC	*Rv2349c*	2627946	cgT/cgC	R251R	Filliol *et al.* 2006: global phylogeny *(Supplementary Table 2)*
Rv2424c		2721562	gcG/gcC	A72A	Filliol *et al.* 2006
Rv2472		2775361	cgC/cgT	R30R	Filliol *et al.* 2006
scoB	*Rv2503c*	2818837	ggT/ggC	G97G	Liu *et al.* 2006
fadD35	*Rv2505c*	2821342	gcG/gcA	A85A	Gutacker *et al.* 2002; Liu *et al.* 2006
Rv2510c		2825581	atA/atC	I(s)503I(s)	Gutacker *et al.* 2002; Filliol *et al.* 2006: global phylogeny *(Supplementary Table 2)*, Beijing specific; Iwamoto *et al.* 2008
fas	*Rv2524c*	2847281	gaT/gaC	D684D	Gutacker *et al.* 2002
Rv2542		2865882	gtT/gtC	V251V	Filliol *et al.* 2006
Rv2567		2891267	ggC/ggT	G491G	Gutacker *et al.* 2002; Filliol *et al.* 2006: global phylogeny *(Supplementary Table 2)*; Gutacker *et al.* 2006: part of 36 SNP-set; Liu *et al.* 2006: part of 36 SNP-set in data set I

Supplementary Table 5: (continued)

Gene	Synonym	Position	Base change	Amino acid	Reference & comment
Rv2578c		2903439	agC/agT	S31S	Gutacker *et al.* 2002; Filliol *et al.* 2006
ruvB	*Rv2592c*	2923391	ccA/ccG	P281P	Filliol *et al.* 2006; Dos Vultos *et al.* 2008
Rv2614A		2943411	ctT/ctC	L12L	Lee *et al.* 2008[b]; Zheng *et al.* 2008; *common to all 3 genomes, error in original H37Rv sequence (Supplementary Table 1)*
Rv2680		2996194	gtT/gtA	V30V	Lee *et al.* 2008[b]; Zheng *et al.* 2008; *common to all 3 genomes, error in original H37Rv sequence (Supplementary Table 1)*
Rv2695		3012293	acA/acG	T126T	Lee *et al.* 2008[b]; Zheng *et al.* 2008; *common to all 3 genomes, error in original H37Rv sequence (Supplementary Table 1)*
PE_PGRS47	*Rv2741*	3054081	ggA/ggG	G56G	Gutacker *et al.* 2002
Rv2795c		3104189	tgT/tgC	C241C	Gutacker *et al.* 2002
Rv2802c		3111476	tcG/tcA	S116S	Gutacker *et al.* 2002; Filliol *et al.* 2006: global phylogeny *(Supplementary Table 3)*, marker for Beijing; Gutacker *et al.* 2006: part of 36 SNP-set; Liu *et al.* 2006: part of 36 SNP-set in data set I
ffh	*Rv2916c*	3226181	cgT/cgG	R35R	Liu *et al.* 2006
ppsB	*Rv2932*	3254365	ctT/ctC	L1098L	Lee *et al.* 2008[b];

Supplementary Table 5: (continued)

Gene	Synonym	Position	Base change	Amino acid	Reference & comment
					Zheng *et al.* 2008; *common to all 3 genomes, error in original H37Rv sequence (Supplementary Table 1)*
ppsC	*Rv2933*	3256494	ggA/ggG	G270G	Gutacker *et al.* 2002; Liu *et al.* 2006
Rv2957		3310174	aaG/aaA	K235K	Hershberg *et al.* 2008
Rv2962c		3314412	gcT/gcC	A237A	Gutacker *et al.* 2002; Liu *et al.* 2006; Hershberg *et al.* 2008
leuB	*Rv2995c*	3352932	acG/acC	T179T	Filliol *et al.* 2006: global phylogeny *(Supplementary Table 3)*; Liu *et al.* 2006
PPE47	*Rv3021c*	3379712	gcC/gcG	A247A	Zheng *et al.* 2008; *common to all 3 genomes (Supplementary Table 1)*
		3379784	ggG/ggT	G223G	Zheng *et al.* 2008; *common to all 3 genomes (Supplementary Table 1)*
Rv3041c		3401871	gcT/gcC	A16A	Gutacker *et al.* 2002; Filliol *et al.* 2006; Liu *et al.* 2006
Rv3077	*atsF*	3440464	cgT/cgG	R308R	Gutacker *et al.* 2002; Filliol *et al.* 2006: global phylogeny *(Supplementary Table 2)*, Beijing specific; Liu *et al.* 2006
lipR	*Rv3084*	3450725	gtT/gtC	V243V	Filliol *et al.* 2006: global phylogeny *(Supplementary Table 2)*
Rv3088		3455686	ctG/ctC	L(s)449L	Gutacker *et al.* 2002; Filliol *et al.* 2006:

Supplementary Table 5: (continued)

Gene	Synonym	Position	Base change	Amino acid	Reference & comment
					global phylogeny *(Supplementary Table 2)*; Gutacker *et al.* 2006: part of 36 SNP-set; Liu *et al.* 2006: part of 36 SNP-set in data set I
cyp141	*Rv3121*	3487108	gcC/gcT	A200A	Filliol *et al.* 2006
Rv3132c	*Rv3132c*	3498198	gaG/gaA	E356E	Hershberg *et al.* 2008
Rv3134c		3500243	gcC/gcG	A169A	Gutacker *et al.* 2002
fadB4	*Rv3141*	3508970	gaA/gaG	E292E	Filliol *et al.* 2006
Rv3183		3550789	gcC/gcG	A25A	Gutacker *et al.* 2002; Filliol *et al.* 2006: global phylogeny *(Supplementary Table 3)*, marker for Beijing Gutacker *et al.* 2006: part of 36 SNP-set; Liu *et al.* 2006: part of 36 SNP-set in data set I
Rv3239c		3614982	ctA/ctG	L874L(s)	Gutacker *et al.* 2002; Filliol *et al.* 2006; Gutacker *et al.* 2006: part of 36 SNP-set; Liu *et al.* 2006: part of 36 SNP-set in data set I
Rv3243c		3622441	gtT/gtG	V217V(s)	Filliol *et al.* 2006; Liu *et al.* 2006
Rv3294c		3675504	atC/atA	I(s)164I(s)	Filliol *et al.* 2006
nei	*Rv3297*	3681548	Agg/Cgg	R77R	Gutacker *et al.* 2002; Liu *et al.* 2006; Dos Vultos *et al.* 2008
Rv3335c		3721806	ggC/ggG	G265G	Filliol *et al.* 2006;

Supplementary Table 5: (continued)

Gene	Synonym	Position	Base change	Amino acid	Reference & comment
					Liu *et al.* 2006
idsA1	*idsA*, *Rv3398c*	3815477	gcC/gcA	A210A	Gutacker *et al.* 2002; Liu *et al.* 2006
guaB3	*Rv3410c*	3829770	ccA/ccG	P47P	Gutacker *et al.* 2002; Filliol *et al.* 2006; Gutacker *et al.* 2006: part of 36 SNP-set; Liu *et al.* 2006: part of 36 SNP-set in data set I
rimI	*Rv3420c*	3838871	gcT/gcC	A64A	Liu *et al.* 2006; Deshayes *et al.* 2008
Rv3440c		3859893	gaG/gaA	E28E	Liu *et al.* 2006
kgtP	*Rv3476c*	3892671	gtT/gtC	V350V	Liu *et al.* 2006
fadE33	*Rv3564*	4005607	Ttg/Ctg	L(s)121L(s)	Gutacker *et al.* 2002; Liu *et al.* 2006
ispF	*Rv3581c*	4024273	gtA/gtG	V25V(s)	Gutacker *et al.* 2002; Filliol *et al.* 2006: global phylogeny *(Supplementary Table 2)*; Liu *et al.* 2006
radA	*Rv3585*	4026899	caG/caA	Q152Q	Liu *et al.* 2006; Dos Vultos *et al.* 2008
ftsH	*Rv3610c*	4050811	taC/taT	Y691Y	Filliol *et al.* 2006
Rv3695		4137829	gcC/gcT	A208A	Filliol *et al.* 2006: global phylogeny *(Supplementary Table 2)*, Beijing specific; Iwamoto *et al.* 2008
gshA	*Rv3704c*	4147070	Ttg/Ctg	L(s)373L(s)	Lee *et al.* 2008[b]; Zheng *et al.* 2008; *common to all 3 genomes, error in original H37Rv sequence (Supplementary*

Supplementary Table 5: (continued)

Gene	Synonym	Position	Base change	Amino acid	Reference & comment
					Table 1)
asd	*Rv3708c*	4151855	ccT/ccC	P121P	Gutacker *et al.* 2002; Filliol *et al.* 2006; Liu *et al.* 2006
dnaZX	*Rv3721c*	4166441	caC/caT	H97H	Dos Vultos *et al.* 2008
Rv3714c		4159195	ccA/ccG	P209P	Gutacker *et al.* 2002; Liu *et al.* 2006
ligC	*Rv3731*	4182387	gtG/gtC	V(s)210V	Dos Vultos *et al.* 2008
Rv3737		4189210	ccG/ccT	P504P	Filliol *et al.* 2006
Rv3777		4222882	ctA/ctG	L63L(s)	Liu *et al.* 2006
embC	*Rv3793*	4242643	cgC/cgT	R927R	Gutacker *et al.* 2002; Ramaswamy *et al.* 2000; Gutacker *et al.* 2006: part of 36 SNP-set; Liu *et al.* 2006: part of 36 SNP-set in data set I; Safi *et al.* 2008: CDC1551
embA	*Rv3794*	4243460	tgC/tgT	C76C	Ramaswamy *et al.* 2000
accD4	*Rv3799c*	4255922	caT/caC	H9H	Gutacker *et al.* 2002; Filliol *et al.* 2006: global phylogeny *(Supplementary Table 2)*; Liu *et al.* 2006
pks13	*Rv3800c*	4257220	cgT/cgC	R1309R	Gutacker *et al.* 2002; Liu *et al.* 2006
Rv3815c		4280708	gtC/gtT	V27V	Filliol *et al.* 2006: global phylogeny *(Supplementary Table 2)*, marker for Beijing; Gagneux *et al.* 2007: marker for Beijing

Supplementary Table 5: (continued)

Gene	Synonym	Position	Base change	Amino acid	Reference & comment
pks2	*Rv3825c*	4296015	gaC/gaT	D1197D	Gutacker *et al.* 2002; Filliol *et al.* 2006; Liu *et al.* 2006
Rv3831		4306155	agC/agT	S133S	Liu *et al.* 2006
bfrB	*Rv3841*	4314645	ctA/ctG	L156L(s)	Gutacker *et al.* 2002; Filliol *et al.* 2006: global phylogeny *(Supplementary Table 3)*; Liu *et al.* 2006; Wang *et al.* 2007: up-regulated in RMP resistant isolate
Rv3871		4350305	ccG/ccA	P493P	Gutacker *et al.* 2002; Filliol *et al.* 2006
Rv3896c		4382054	gcA/gcG	A266A	Liu *et al.* 2006

[a]Present in both Beijing isolates but not H37Rv.

[b]Our thanks to Roland Krause from the Department of Computational Molecular Biology (Max Planck Institute for Molecular Genetics) for translating the H37Ra positions into the corresponding one in H37Rv.

[c]Please note that this corresponds to amino acid 10**81** based on the TIGR annotation of H37Rv in which the N-terminus was annotated to start 6 amino acids upstream of the start in TubercuList.

Supplementary Table 6: **Previously described non-synonymous SNPs.** Unless otherwise stated in *italic font*, they were part of the Beijing K-family backbone[a] of both isolates. Comments were taken from the publications in question. Mutations causing drug resistance are listed separately in Table 1.

Gene	Synonym	Position	Base change	Amino acid	Reference & comment
recF	*Rv0003*	4013	aTc/aCc	I(s)245T	Gutacker *et al.* 2006; Dos Vultos *et al.* 2008
gyrA	*Rv0006*	7362	Gag/Cag	E21Q	Sekiguchi *et al.* 2007: association with fluoroquinolone resistance unclear; Hershberg *et al.* 2008
		7585	aGc/aCc	S95T	Sreevatsan *et al.* 1997: principal genetic group 1; Alland *et al.* 2003: no apparent selective advantage; Cheng *et al.* 2004: no impact on fluoroquinolone susceptibility; Filliol *et al.* 2006; Gutacker *et al.* 2006; Sekiguchi *et al.* 2007; Hershberg *et al.* 2008
		9304	gGc/gAc	G668D	Gutacker *et al.* 2006; Hershberg *et al.* 2008
Rv0012		14785	Tgc/Cgc	C233R	Lee *et al.* 2008[b]; Zheng *et al.* 2008; *common to all 3 genomes (Supplementary Table 1)*
ino1	*tbINO*, *Rv0046c*	50557	Agg/Ggg	R190G	Gutacker *et al.* 2006
Rv0048c		51949	gTc/gCc	V250A	Gutacker *et al.* 2006
Rv0064		69989	gGt/gAt	G457D	Frigui *et al.* 2008; Lee *et al.* 2008[b]; Zheng *et al.* 2008; *common to all 3 genomes (Supplementary Table 1)*
		70267	Gtc/Ttc	V550F	Gutacker *et al.* 2006
Rv0068		75940	Gtg/Ctg	V(s)214L(s)	Gutacker *et al.* 2006

Supplementary Table 6: (continued)

Gene	Synonym	Position	Base change	Amino acid	Reference & comment
Rv0082		90144	cAa/cGa	Q74R	Lee *et al.* 2008[b]; Zheng *et al.* 2008; *common to all 3 genomes, error in original H37Rv sequence (Supplementary Table 1)*
Rv0095c		104941	CAg/GGg	Q92G	Liu *et al.* 2006: inter-genic at distance 368 in IRMT0105 in data set II; Deshayes *et al.* 2008
		104942	CAg/GGg	Q92G	Liu *et al.* 2006: inter-genic at distance 369 in IRMT0105 in data set II; Deshayes *et al.* 2008
		104944	gCc/gTc	A91V	Liu *et al.* 2006: inter-genic at distance 370 in IRMT0105 in data set II; Deshayes *et al.* 2008
		104962	gCg/gTg	A85V(s)	Liu *et al.* 2006: inter-genic at distance 389 in IRMT0105 in data set II
		105007	aGc/aCc	S70T	Liu *et al.* 2006: inter-genic at distance 434 in IRMT0105 in data set II
		105045	gaC/gaG	D57E	Liu *et al.* 2006: inter-genic at distance 472 in IRMT0105 in data set II
		105139	gGg/gTg	G26V(s)	Gutacker *et al.* 2002; Filliol *et al.* 2006; Liu *et al.* 2006: inter-genic at distance 566 in IRMT0105 in

Supplementary Table 6: (continued)

Gene	Synonym	Position	Base change	Amino acid	Reference & comment
					data set II; Alland *et al.* 2007: global phylogeny *(Supplementary Table 4)*, marker for Beijing
PE_PGRS1	*Rv0109*	132417	Cgc/Ggc	R346G	Lee *et al.* 2008[b]; Zheng *et al.* 2008; *common to all 3 genomes, error in original H37Rv sequence (Supplementary Table 1)*
PE3	*Rv0159c*	188800	Acg/Gcg	T14A	Gutacker *et al.* 2006
mce1F	*Rv0174*	206339	cTg/cCg	L(s)370P	Gutacker *et al.* 2006
Rv0193c		225323	Aag/Gag	K417E	Gutacker *et al.* 2006
Rv0194		227098	aTg/aCg	M(s)74T	Gutacker *et al.* 2006
Rv0204c		242299	Gtg/Ctg	V(s)306L(s)	Lee *et al.* 2008[b]; Zheng *et al.* 2008; *common to all 3 genomes, error in original H37Rv sequence (Supplementary Table 1)*
Rv0259c		310973	gCg/gTg	A182V(s)	Filliol *et al.* 2006
Rv0284		346275	cCg/cGg	P214R	Gutacker *et al.* 2006
Rv0323c		390828	Agt/Ggt	S142G	Frigui *et al.* 2008; Zheng *et al.* 2008; *common to all 3 genomes (Supplementary Table 1)*
Rv0325		392261	Tag/Cag	*75Q	Deshayes *et al.* 2008: not full length in H37Rv and CDC1551
Rv0338c		403980	gCc/gTc	A621V	Gutacker *et al.* 2006; Filliol *et al.* 2006
		404326	Agg/Ggg	R506G	Gutacker *et al.* 2006
PPE8	*Rv0355c*	426909	Tgg/Ggg	W2591G	Lee *et al.* 2008[b];

Supplementary Table 6: (continued)

Gene	Synonym	Position	Base change	Amino acid	Reference & comment
					Zheng *et al.* 2008; *common to all 3 genomes, error in original H37Rv sequence (Supplementary Table 1)*
PPE9	*Rv0388c*	467526	gGg/gCg	G159A	Zheng *et al.* 2008; *common to all 3 genomes (Supplementary Table 1)*
		467546	gaC/gaG	D152E	Zheng *et al.* 2008 *common to all 3 genomes, error in original H37Rv sequence (Supplementary Table 1)*
		467557	Ttg/Gtg	L(s)149V(s)	Zheng *et al.* 2008; *common to all 3 genomes (Supplementary Table 1)*
		467564	caT/caG	H146Q	Zheng *et al.* 2008; *common to all 3 genomes (Supplementary Table 1)*
		467585	caC/caG	H139Q	Zheng *et al.* 2008 *common to all 3 genomes, error in original H37Rv sequence (Supplementary Table 1)*
		467590	Acg/Gcg	T138A	Zheng *et al.* 2008 *common to all 3 genomes, error in original H37Rv sequence (Supplementary Table 1)*
		467638	Cag/Aag	Q122K	Lee *et al.* 2008[b]; Zheng *et al.* 2008; *common to all 3 genomes, error in original H37Rv sequence (Supplementary Table 1)*
Rv0395		475178	gTc/gCc	V80A	Deshayes *et al.* 2008
fadD30	*Rv0404*	484596	cCg/cTg	P207L(s)	Gutacker *et al.* 2006

Supplementary Table 6: (continued)

Gene	Synonym	Position	Base change	Amino acid	Reference & comment
lpqM	*Rv0419*	505974	Gcg/Acg	A297T	Gutacker *et al.* 2006
pssA	*Rv0436c*	524891	gGc/gTc	G167V	Filliol *et al.* 2006
PPE10	*Rv0442c*	532097	Aaa/Gaa	K40E	Lee *et al.* 2008[b]; Zheng *et al.* 2008; *common to all 3 genomes, error in original H37Rv sequence (Supplementary Table 1)*
Rv0473		563577	aAa/aGa	K5R	Lee *et al.* 2008[b]; Zheng *et al.* 2008; *common to all 3 genomes, error in original H37Rv sequence (Supplementary Table 1)*
mmpL2	*Rv0507*	598475	cGc/cAc	R426H	Gutacker *et al.* 2006
PE_PGRS6	*Rv0532*	623508	gCc/gGc	A239G	Lee *et al.* 2008[b]; Zheng *et al.* 2008; *common to all 3 genomes, error in original H37Rv sequence (Supplementary Table 1)*
Rv0538		630722	cGg/cCg	R228P	Filliol *et al.* 2006
pitA	*Rv0545c*	637319	Cct/Tct	P49S	Gutacker *et al.* 2006
recD	*Rv0629c*	721373	gaA/gaC	E120D	Dos Vultos *et al.* 2008: predicted to be non-significant
Rv0658c		754186	cTc/cCc	L75P	Zheng *et al.* 2008
PE_PGRS9	*Rv0746*	836658	Acc/Gcc	T320A	Zheng *et al.* 2008; *common to all 3 genomes (Supplementary Table 1)*
		837033	Acc/Gcc	T445A	Zheng *et al.* 2008; *common to all 3 genomes, error in original H37Rv sequence (Supplementary*

Supplementary Table 6: (continued)

Gene	Synonym	Position	Base change	Amino acid	Reference & comment
					Table 1)
fadA	*Rv0859*	955524	Agc/Ggc	S150G	Deshayes *et al.* 2008
phoR	*Rv0758*	852910	cCc/cTc	P172L	Gutacker *et al.* 2006
Rv0812	*pabC*	906857	atA/atG	I145M	Filliol *et al.* 2006
Rv0881		979704	Ggc/Cgc	G115R	Liu *et al.* 2006; Deshayes *et al.* 2008
Rv0890c		990001	Ccc/Gcc	P866A	Lee *et al.* 2008[b]; Zheng *et al.* 2008; *common to all 3 genomes (Supplementary Table 1)*
Rv0918		1024346	Agt/Ggt	S46G	Gutacker *et al.* 2006
pstA1	*Rv0930*	1037911	Cga/Tga	R305*	Lee *et al.* 2008[b]; Zheng *et al.* 2008; *common to all 3 genomes (Supplementary Table 1)*
Rv0938		1047165	Tgc/Cgc	C344R	Liu *et al.* 2006; Dos Vultos *et al.* 2008
Rv0958		1070702	Tcc/Ccc	S274P	Gutacker *et al.* 2006
Rv0962c		1074558	cCc/cTc	P186L	Gutacker *et al.* 2006
Rv0964c		1076309	Cct/Act	P124T	Gutacker *et al.* 2006
Rv0966c		1077312	gTg/gCg	V(s)175A	Frigui *et al.* 2008; Zheng *et al.* 2008
pepD	*mtb32b*, *Rv0983*	1100234	cTg/cCg	L(s)390P	Gutacker *et al.* 2006
Rv0990c		1107940	Tcc/Gcc	S54A	Gutacker *et al.* 2006
metS	*Rv1007c*	1126889	Cgc/Ggc	R39G	Gutacker *et al.* 2006
tatD	*yjjV*, *Rv1008*	1127648	aCc/aAc	T187N	Gutacker *et al.* 2006
glyA1	*glyA*, *Rv1093*	1220680	gTa/gCa	V36A	Gutacker *et al.* 2006
mutT2	*Rv1160*	1286766	Gga/Cga	G58R	Rad *et al.* 2003: Beijing specific; Lari *et al.* 2006:

Supplementary Table 6: (continued)

Gene	Synonym	Position	Base change	Amino acid	Reference & comment
					Beijing specific, not associated with drug resistance, located near active site; Dos Vultos *et al.* 2008: predicted to be significant; Hershberg *et al.* 2008; Iwamoto *et al.* 2008; Rindi *et al.* 2008: Beijing specific, subsequent to RD181 *(Table 1)*; Moreland *et al.* 2009: leads to reduced affinity for divalent cations
pks3	*Rv1180*	1315191	taA/taC	*489Y	Dubey *et al.* 2002: fuses *pks3* and *pks4* into *msl3*; Deshayes *et al.* 2008: specific to H37Rv; Frigui *et al.* 2008; Lee *et al.* 2008[b]; Zheng *et al.* 2008; *common to all 3 genomes (Supplementary Table 1)*
Rv1186c		1328687	Ccc/Gcc	P207A	Gutacker *et al.* 2002
PPE18	*mtb39a*, *Rv1196*	1340208	cGg/cAg	R287Q	Hebert *et al.* 2007: most common SNP
pknH	*Rv1266c*	1414021	cGg/cAg	R607Q	Frigui *et al.* 2008; Lee *et al.* 2008[b]; Zheng *et al.* 2008; *common to all 3 genomes (Supplementary Table 1)*
alkA	ada, *Rv1317c*	1479085	Atc/Gtc	I(s)12V	Nouvel *et al.* 2007; Dos Vultos *et al.* 2008: predicted to be significant
Rv1320c		1482627	Acg/Gcg	T531A	Nouvel *et al.* 2007
Rv1321		1484708	Agc/Cgc	S144R	Nouvel *et al.* 2007

Supplementary Table 6: (continued)

Gene	Synonym	Position	Base change	Amino acid	Reference & comment
Rv1374c		1547125	Aca/Gca	T136A	Gutacker *et al.* 2006
PE_PGRS25	*Rv1396c*	1573660	agA/agC	R66S	Filliol *et al.* 2006
lipI	*Rv1400c*	1576481	Acc/Ccc	T106P	Gutacker *et al.* 2002; Filliol *et al.* 2006
		1576527	ttC/ttA	F90L	Gutacker *et al.* 2002; Filliol *et al.* 2006; Gutacker *et al.* 2006: part of 36 SNP-set; Liu *et al.* 2006: part of 36 SNP-set in data set I
uvrC	*Rv1420*	1594906	Gtc/Atc	V289I(s)	Dos Vultos *et al.* 2008: double codon mutation specific to Beijing; predicted to be non-significant
		1595342	gTg/gCg	V(s)434A	Dos Vultos *et al.* 2008: double codon mutation specific to Beijing predicted to be non-significant
Rv1431		1608276	aAc/aCc	N65T	Gutacker *et al.* 2006
opcA	*Rv1446c*	1624791	cGa/cCa	R192P	Gutacker *et al.* 2006; Jiang *et al.* 2006: up-regulated in INH resistant isolates; Wang *et al.* 2007: down-regulated in RMP resistant isolate
tkt	*Rv1449c*	1630148	Tac/Gac	Y18D	Filliol *et al.* 2006
Rv1453		1639594	cCa/cAa	P405Q	Gutacker *et al.* 2006
Rv1463		1651308	gAa/gGa	E198G	Gutacker *et al.* 2006
Rv1498c		1689349	cGc/cAc	R191H	Gutacker *et al.* 2006

Supplementary Table 6: (continued)

Gene	Synonym	Position	Base change	Amino acid	Reference & comment
frdC	*Rv1554*	1760292	Atg/Gtg	M(s)40V(s)	Deshayes *et al.* 2008
Rv1592c		1792777	Att/Gtt	I(s)322V	Ramaswamy *et al.* 2003: observed in all group 1, some group 2 but not in any group 3 isolates
hisH	*Rv1602*	1803265	aGc/aAc	S201N	Betts *et al.* 2000: possibly responsible for absence of *hisA* on protein level in CDC1551
impA	*Rv1604*	1804409	cCa/cAa	P124Q	Gutacker *et al.* 2006
polA	*Rv1629*	1831220	Acc/Ccc	T186P	Dos Vultos *et al.* 2008: Beijing specific, predicted to be significant
		1831226	Agg/Ggg	R188G	Dos Vultos *et al.* 2008: Beijing specific, predicted to be significant
tsnR	*Rv1644*	1854300	cTg/cCg	L(s)232P	Gutacker *et al.* 2006
pks8	*Rv1662*	1885772	Gcc/Acc	A1357T	Gutacker *et al.* 2006
Rv1733c		1960284	caG/caT	Q68H	Hershberg *et al.* 2008
Rv1783		2020563	tAg/tTg	*436L(s)	Deshayes *et al.* 2008: specific to H37Rv; Lee *et al.* 2008[b]; Zheng *et al.* 2008; *common to all 3 genomes, error in original H37Rv sequence (Supplementary Table 1)*
PPE31	*Rv1807*	2049065	tTc/tCc	F223S	Gutacker *et al.* 2006
		2049097	Gtc/Ctc	V234L	Gutacker *et al.* 2006
PPE33	*Rv1809*	2052035	Gtg/Ttg	V(s)252L(s)	Gutacker *et al.* 2006; Filliol *et al.* 2006
Rv1812c		2055271	cTa/cCa	L30P	Gutacker *et al.* 2006
Rv1815		2057774	Atc/Ttc	I(s)83F	Frigui *et al.* 2008;

Supplementary Table 6: (continued)

Gene	Synonym	Position	Base change	Amino acid	Reference & comment
					Lee *et al.* 2008[b]; Zheng *et al.* 2008; *common to all 3 genomes (Supplementary Table 1)*
Rv1835c		2082053	Tgc/Cgc	C179R	Gutacker *et al.* 2002
apa	*modD*, *mpt32*, *Rv1860*	2108141	Ttc/Ctc	F136L	Musser *et al.* 2000: not found in group 3 isolates; Filliol *et al.* 2006
katG	*Rv1908c*	2154724	cGg/cTg	R463L(s)	Sreevatsan, S. *et al.* 1997: principal genetic group 1; van Doorn *et al.* 2001: not associated with INH resistance; Ramaswamy *et al.* 2003; Filliol *et al.* 2006; Gutacker *et al.* 2006; Liu *et al.* 2006; Alland *et al.* 2007: global phylogeny *(Supplementary Table 4)*
PPE34	*Rv1917c*	2165286	Tca/Gca	S676A	Gutacker *et al.* 2006
Rv2024c		2269780	gAc/gGc	D154G	Gutacker *et al.* 2006
fadD31	Rv1925	2177654	Atg/Ctg	M(s)190L(s)	Zheng *et al.* 2008 *common to all 3 genomes, error in original H37Rv sequence (Supplementary Table 1)*
Rv1979c		2221796	Gtc/Atc	V457I(s)	Lee *et al.* 2008[b]; Zheng *et al.* 2008; *common to all 3 genomes, error in original H37Rv sequence (Supplementary Table 1)*
Rv2017		2264782	gCg/gAg	A262E	Filliol *et al.* 2006
Rv2037c		2282787	tGt/tAt	C312Y	Frigui *et al.* 2008; Lee *et al.* 2008[b];

Supplementary Table 6: (continued)

Gene	Synonym	Position	Base change	Amino acid	Reference & comment
					Zheng *et al.* 2008; *common to all 3 genomes (Supplementary Table 1)*
pks12	*Rv2048c*	2296042	Ccg/Gcg	P3649A	Gutacker *et al.* 2006
		2297976	tCa/tTa	S3004L	Lee *et al.* 2008[b]; Zheng *et al.* 2008; *common to all 3 genomes, error in original H37Rv sequence (Supplementary Table 1)*
helZ	*Rv2101*	2361623	Atg/Ctg	M(s)462L(s)	Lee *et al.* 2008[b]; Zheng *et al.* 2008; *common to all 3 genomes, error in original H37Rv sequence (Supplementary Table 1)*
Rv2079		2335494	tAc/tGc	Y47C	Gutacker *et al.* 2006
Rv2082		2340621	cCc/cGc	P638R	Gutacker *et al.* 2006
prcA	*Rv2109c*	2369326	cGc/cCc	R135P	Gutacker *et al.* 2002; Filliol *et al.* 2006; Gutacker *et al.* 2006: part of 36 SNP-set; Liu *et al.* 2006: part of 36 SNP-set in data set I
aroG	*Rv2178c*	2440926	gaC/gaA	D265E	Gutacker *et al.* 2006
fadD15	*Rv2187*	2448458	aCc/aTc	T100I(s)	Gutacker *et al.* 2006
Rv2226		2499726	Gat/Aat	D299N	Gutacker *et al.* 2006
accD6	*Rv2247*	2521428	gAc/gGc	D229G	Ramaswamy *et al.* 2003: not involved in INH resistence
Rv2307c		2578626	aTg/aCg	M(s)24T	Filliol *et al.* 2006
rpfE	*Rv2450c*	2751804	cGg/cAg	R126Q	Lee *et al.* 2008[b]; Hershberg *et al.* 2008;

Supplementary Table 6: (continued)

Gene	Synonym	Position	Base change	Amino acid	Reference & comment
					Zheng *et al.* 2008; *common to all 3 genomes (Supplementary Table 1)*
		2752122	aCg/aGg	T20R	Hershberg *et al.* 2008
plsB2	*Rv2482c*	2786952	Tgc/Cgc	C778R	Gutacker *et al.* 2006; Filliol *et al.* 2006
pdhC	*Rv2495c*	2809621	Acc/Gcc	T107A	Frigui *et al.* 2008; Lee *et al.* 2008[b]; Zheng *et al.* 2008; *common to all 3 genomes (Supplementary Table 1)*
fas	*Rv2524c*	2841022	Tgc/Cgc	C2771R	Gutacker *et al.* 2006
Rv2560		2880702	Gtc/Ctc	V210L	Filliol *et al.* 2006
Rv2627c		2954439	Aga/Gga	R104G	Lee *et al.* 2008[b]; Hershberg *et al.* 2008; Zheng *et al.* 2008; *common to all 3 genomes (Supplementary Table 1)*
Rv2629		2955957	gAt/gCt	D64A	Wang *et al.* 2007: up-regulated in RMP resistant isolate, associated with RMP resistance; Chakravorty *et al.* 2008: **not** associated with RMP resistance but Beijing marker; Homolka *et al.* 2009: **not** associated with RMP resistance but Beijing marker; Louw *et al.* 2009: **not** associated with RMP resistance but Beijing marker
ppgK	*Rv2702*	3017465	aTa/aCa	I(s)203T	Gutacker *et al.* 2006
recX	*Rv2736c*	3048912	Gtt/Ctt	V59L	Gutacker *et al.* 2006; Dos Vultos *et al.* 2008: predicted to be non-

Supplementary Table 6: (continued)

Gene	Synonym	Position	Base change	Amino acid	Reference & comment
					significant
hsdM	*Rv2756c*	3069167	cTg/cCg	L(s)306P	Gutacker *et al.* 2002; Gutacker *et al.* 2006; Filliol *et al.* 2006; Liu *et al.* 2006
PE27	*Rv2769c*	3078178	Gtg/Atg	V(s)270M(s)	Gutacker *et al.* 2006
ppe44	*Rv2770c*	3079877	tTc/tCc	F194S	Gutacker *et al.* 2006; Rindi *et al.* 2007: Beijing specific
Rv2771c		3080795	cTc/cCc	L80P	Gutacker *et al.* 2006
Rv2812		3118000	Agg/Ggg	R395G	Gutacker *et al.* 2006
dipZ	*Rv2874*	3186860	Tat/Gat	Y672D	Gutacker *et al.* 2006
Rv2896c		3205978	Tcg/Gcg	S153A	Lee *et al.* 2008[b]; Zheng *et al.* 2008; *common to all 3 genomes, error in original H37Rv sequence (Supplementary Table 1)*
ppsA	*Rv2931*	3249025	cTa/cGa	L1194R	Gutacker *et al.* 2006
mas	*Rv2940c*	3276703	Act/Cct	T2005P	Gutacker *et al.* 2006
Rv2952		3304966	Ggg/Agg	G176R	Hershberg *et al.* 2008
Rv2979c		3335708	cCg/cGg	P14R	Dos Vultos *et al.* 2008: predicted to be non-significant
gpdA2	*gpsA*, *Rv2982c*	3338603	Ccg/Gcg	P133A	Gutacker *et al.* 2006
gatA	*Rv3011c*	3370177	Atg/Ctg	M(s)420L(s)	Lee *et al.* 2008[b]; Zheng *et al.* 2008; *common to all 3 genomes, error in original H37Rv sequence (Supplementary Table 1)*
PPE47	*Rv3021c*	3379708	Ctg/Gtg	L(s)249V(s)	Zheng *et al.* 2008; *common to all 3 genomes*

Supplementary Table 6: (continued)

Gene	Synonym	Position	Base change	Amino acid	Reference & comment
					(Supplementary Table 1)
		3379788	gGg/gCg	G222A	Zheng *et al.* 2008; *common to all 3 genomes (Supplementary Table 1)*
ligB	*Rv3062*	3425854	Ccg/Tcg	P91S	Dos Vultos *et al.* 2008: predicted to be non-significant
cstA	*Rv3063*	3428917	Cgt/Agt	R559S	Gutacker *et al.* 2006
Rv3077	*atsF*	3440468	Ggc/Cgc	G310R	Gutacker *et al.* 2006
lipY	*PE_PGRS63* *Rv3097c*	3466919	gGc/gCc	G58A	Gutacker *et al.* 2006
Rv3113		3480474	gGa/gAa	G134E	Gutacker *et al.* 2006
sugI	*Rv3331*	3718357	cCg/cTg	P423L(s)	Zheng *et al.* 2008; *common to all 3 genomes (Supplementary Table 1)*
Rv3137		3503895	cCg/cTg	P168L(s)	Gutacker *et al.* 2006
PPE52	*Rv3144c*	3510642	Agc/Ggc	S226G	Lee *et al.* 2008[b]; Zheng *et al.* 2008; *common to all 3 genomes, error in original H37Rv sequence (Supplementary Table 1)*
Rv3190c		3556275	cTg/cCg	L(s)138P	Gutacker *et al.* 2006
nudC	*Rv3199c*	3571828	cCg/cGg	P239R	Dos Vultos *et al.* 2008
idsB	*Rv3383c*	3798095	gTc/gGc	V132G	Gutacker *et al.* 2006; Liu *et al.* 2006
Rv3407		3826501	Cgc/Tgc	R84C	Gutacker *et al.* 2006; Hershberg *et al.* 2008
esxU	*Rv3445c*	3863138	Ccg/Tcg	P63S	Gutacker *et al.* 2006
Rv3447c		3864995	Agc/Ggc	S1082G	Gutacker *et al.* 2006
Rv3479		3896340	cTc/cGc	L174R	Lee *et al.* 2008[b]; Zheng *et al.* 2008;

Supplementary Table 6: (continued)

Gene	Synonym	Position	Base change	Amino acid	Reference & comment
					common to all 3 genomes (Supplementary Table 1)
nth	*Rv3674c*	4115890	cCc/cGc	P2R	Gutacker *et al.* 2006; Dos Vultos *et al.* 2008: Beijing specific, predicted to be non-significant
dnaQ	*Rv3711c*	4156099	Gtg/Ttg	V(s)211L(s)	Gutacker *et al.* 2002; Filliol *et al.* 2006; Liu *et al.* 2006; Dos Vultos *et al.* 2008
		4156503	gGc/gAc	G76D	Dos Vultos *et al.* 2008
recR	*Rv3715c*	4160371	Ggt/Tgt	G44C	Dos Vultos *et al.* 2008
ligC	*Rv3731*	4182695	cGc/cAc	R313H	Dos Vultos *et al.* 2008
Rv3839		4313128	Ccc/Tcc	P122S	Gutacker *et al.* 2006
PE35	*Rv3872*	4351039	Gaa/Taa	E99*	Hershberg *et al.* 2008
Rv3894c		4378504	gAt/gGt	D650G	Gutacker *et al.* 2006
		4379680	cGc/cCc	R258P	Gutacker *et al.* 2006
Rv3898c		4383655	Tag/Cag	*111Q	Deshayes *et al.* 2008: not full length in H37Rv and CDC1551
Rv3908		4393590	Cgg/Ggg	R48G	Rad *et al.* 2003: Beijing specific; Lari *et al.* 2006: Beijing specific, not associated with drug resistance; Dos Vultos *et al.* 2008: predicted to be non-significant; Iwamoto *et al.* 2008; Hershberg *et al.* 2008; Rindi *et al.* 2008: Beijing specific, subsequent

Supplementary Table 6: (continued)

Gene	Synonym	Position	Base change	Amino acid	Reference & comment
					to RD181 *(Table 1)*
gid	*gidB*, *Rv3919c*	4407904	tCt/tTt	S100F	Zheng *et al.* 2008; *common to all 3 genomes, error in original H37Rv sequence (Supplementary Table 1)*
		4407927	gaA/gaC	E92D	Okamoto *et al.* 2007: not involved in SM resistance; Spies *et. al* 2008

[a]Present in both Beijing isolates but not H37Rv.

[b]Our thanks to Roland Krause from the Department of Computational Molecular Biology (Max Planck Institute for Molecular Genetics) for translating the H37Ra positions into the corresponding one in H37Rv.

Supplementary Table 7: **Previously described inter-genic SNPs.** Unless otherwise stated in *italic font*, they were part of the Beijing K-family backbone[a] of both isolates. Comments were taken from the publications in question.

Inter-genic location	Position	Base change	Distance	Reference & comment
dnaA-dnaN	1977	A/G	453, 75	Filliol *et al.* 2006; Gutacker *et al.* 2006; Liu *et al.* 2006; Alland *et al.* 2007: global phylogeny *(Supplementary Table 4)*
alaT-Rv0008c	11820	C/G	636, 54	Liu *et al.* 2006
Rv0021c-whiB5	26959	C/G	78, 64	Gutacker *et al.* 2006; Liu *et al.* 2006
Rv0067c-Rv0068	75233	C/A	35, 68	Liu *et al.* 2006
Rv0110-Rv0111	133839	C/T	70, 111	Gutacker *et al.* 2006; Liu *et al.* 2006
Rv0383c-clpB	459399	A/C	84, 57	Lee *et al.* 2008[b]; *common to all 3 genomes, error in original H37Rv sequence (Supplementary Table 1)*
thiG-lpqL	503354	G/C	230, 142	Gutacker *et al.* 2006; Liu *et al.* 2006
Rv0474-hbhA	565655	A/G	212, 142	Gutacker *et al.* 2006; Liu *et al.* 2006
Rv0517-Rv0518	610120	T/G	64, 68	Gutacker *et al.* 2006; Liu *et al.* 2006
Rv0681-rpsL	781395	T/C	84, 165	Gutacker *et al.* 2006; Liu *et al.* 2006
Rv0749A-Rv0750	842030	C/T	156, 3	Liu *et al.* 2006
Rv0927c-pstS3	1034758	C/T	127, 145	Jiang *et al.* 2007: leads to loss of expression of *Rv0927c*, Beijing specific
lprP-Rv0963c	1075279	T/C	165, 18	Gutacker *et al.* 2006; Liu *et al.* 2006

Supplementary Table 7: (continued)

Inter-genic location	Position	Base change	Distance	Reference & comment
PE_PGRS18-mprA	1096567	A/G	116, 249	Gutacker *et al.* 2006: corresponds to inter-genic SNP at distance 114 in paper; Liu *et al.* 2006
	1096633	T/G	182, 183	Gutacker *et al.* 2006: corresponds to inter-genic SNP at distance 180 in paper; Liu *et al.* 2006
PE8-Rv1041c	1164336	G/A	960, 236	Deshayes *et al.* 2008
Rv1075c-lipU	1200418	A/G	48, 349	Gutacker *et al.* 2006; Liu *et al.* 2006
phoH2-Rv1096	1224367	T/C	69, 18	Filliol *et al.* 2006; Gutacker *et al.* 2006; Liu *et al.* 2006
Rv1227c-lpqX	1370852	T/G	27, 68	Filliol *et al.* 2006
Rv1264-Rv1265	1413148	C/T	61, 112	Gutacker *et al.* 2006; Liu *et al.* 2006
murA-rrs	1471659	C/T	82, 187	Bhargava *et al.* 1990; Gutacker *et al.* 2006; Nouvel *et al.* 2007; Lee *et al.* 2008[b]; Zheng *et al.* 2008; *common to all 3 genomes (Supplementary Table 1)*
pks5-papA4	1728837	A/G	428, 116	Gutacker *et al.* 2006; Liu *et al.* 2006
coaE-Rv1632c	1836286	G/C	50, 101	Gutacker *et al.* 2006; Liu *et al.* 2006
PPE23-Rv1706A	1933988	G/A	110, 494	Gutacker *et al.* 2006; Liu *et al.* 2006
Rv1829-Rv1830	2074754	C/T	317, 87	Filliol *et al.* 2006; Gutacker *et al.* 2006; Liu *et al.* 2006
fbpB-Rv1887	2135870	T/C	3, 388	Musser *et al.* 2000:

Supplementary Table 7: (continued)

Inter-genic location	Position	Base change	Distance	Reference & comment
				found in all isolates of principal genetic group 3 only
PPE34-PPE35	2167489	T/C	178, 160	Zheng *et al.* 2008; *common to all 3 genomes, error in original H37Rv sequence (Supplementary Table 1)*
Rv1979c-mpt64	2223293	T/C	129, 50	Gutacker *et al.* 2006; Liu *et al.* 2006
cfp21-Rv1985c	2228967	A/G	406, 24	Filliol *et al.* 2006
Rv2005c-otsB1	2251999	A/G	116, 3	Gutacker *et al.* 2006; Liu *et al.* 2006; Lee *et al.* 2008[b]; Zheng *et al.* 2008; *common to all 3 genomes (Supplementary Table 1)*
Rv2017-Rv2018	2265059	T/G	T/G	Gutacker *et al.* 2006; Liu *et al.* 2006
PE_PGRS38-pbpB	2424925	A/G	87, 123	Gutacker *et al.* 2006; Liu *et al.* 2006
Rv2242-fabD	2516567	G/C	19, 220	Gutacker *et al.* 2006; Liu *et al.* 2006
nadD-Rv2422	2718852	T/G	44, 231	Gutacker *et al.* 2006; Lee *et al.* 2008[b]; Zheng *et al.* 2008; *common to all 3 genomes (Supplementary Table 1)*
Rv2451-Rv2452c	2752698	C/A	38, 150	Gutacker *et al.* 2006; Liu *et al.* 2006
Rv2779c-ald	3086788	T/C	34, 32	Gutacker *et al.* 2006; Liu *et al.* 2006
Rv2955c-Rv2956	3308606	G/A	61, 62	Gutacker *et al.* 2006; Liu *et al.* 2006

Supplementary Table 7: (continued)

Inter-genic location	Position	Base change	Distance	Reference & comment
Rv3241c-Rv3242c	3621423	A/G	169, 147	Liu *et al.* 2006
PPE60-Rv3479	3895727	C/A	120, 93	Gutacker *et al.* 2006; Deshayes *et al.* 2008
Rv3618-esxV	4059904	A/G	19, 80	Gutacker *et al.* 2006; Liu *et al.* 2006
Rv3661-Rv3662c	4100975	T/C	465, 290	Zheng *et al.* 2008
whiB6-Rv3863	4338732	G/A	211, 117	Gutacker *et al.* 2006; Liu *et al.* 2006
Rv3898c-Rv3899c	4384007	C/G	22, 140	Deshayes *et al.* 2008

[a]Present in both Beijing isolates but not H37Rv.

[b]Our thanks to Roland Krause from the Department of Computational Molecular Biology (Max Planck Institute for Molecular Genetics) for translating the H37Ra positions into the corresponding one in H37Rv.

References for SNPs and deletions

Alland, D. *et al.* Modeling bacterial evolution with comparative-genome-based marker systems: application to Mycobacterium tuberculosis evolution and pathogenesis. *J. Bacteriol.* **185**, 3392-9 (2003)

Alland, D. *et al.* Role of large sequence polymorphisms (LSPs) in generating genomic diversity among clinical isolates of Mycobacterium tuberculosis and the utility of LSPs in phylogenetic analysis. *J. Clin. Microbiol.* **45**, 39-46 (2007)

Baker, L., Brown, T., Maiden, M. C. & Drobniewski, F. Silent nucleotide polymorphisms and a phylogeny for Mycobacterium tuberculosis. *Emerg. Infect. Dis.* **10**, 1568-77 (2004)

Betts, J. C. *et al.* Comparison of the proteome of Mycobacterium tuberculosis strain H37Rv with clinical isolate CDC 1551. *Microbiology* **146**, 3205-16 (2000)

Bhargava, S., Tyagi, A. K. & Tyagi, J. S. tRNA genes in mycobacteria: organization and molecular cloning. *J. Bacteriol.* **172**, 2930-4 (1990)

Chakravorty, S. *et al.* Rifampin-resistance, Beijing-W clade/SNP cluster group two phylogeny and the Rv2629 191-C allele in Mycobacterium tuberculosis strains. *J. Clin. Microbiol.* **46**, 2555-60 (2008)

Cheng, A. F. *et al.* Multiplex PCR amplimer conformation analysis for rapid detection of gyrA mutations in fluoroquinolone-resistant Mycobacterium tuberculosis clinical isolates. *Antimicrob. Agents Chemother.* **48**, 596-601 (2004)

Deshayes, C. *et al.* Detecting the molecular scars of evolution in the Mycobacterium tuberculosis complex by analyzing interrupted coding sequences. *BMC Evol. Biol.* **8**, 78 (2008)

Dos Vultos, T. *et al.* Evolution and Diversity of Clonal Bacteria: The Paradigm of Mycobacterium tuberculosis. *PLoS ONE* **3**, e1538 (2008)

Dubey, V. S. Sirakova, T. D. & Kolattukudy, P. E. Disruption of msl3 abolishes the synthesis of mycolipanoic and mycolipenic acids required for polyacyltrehalose synthesis in Mycobacterium tuberculosis H37Rv and causes cell aggregation. *Mol. Microbiol.* **45**, 1451-9 (2002)

Filliol, I. *et al.* Global phylogeny of Mycobacterium tuberculosis based on single nucleotide polymorphism (SNP) analysis: insights into tuberculosis evolution, phylogenetic accuracy of other DNA fingerprinting systems, and recommendations for a minimal standard SNP set. *J. Bacteriol.* **188**, 759-72 (2006)

Frigui, W. *et al.* Control of M. tuberculosis ESAT-6 secretion and specific T cell recognition by PhoP. *PLoS Pathog.* **8**, e33 (2008)

Gagneux, S. & Small, P.M. Global phylogeography of Mycobacterium tuberculosis and implications for tuberculosis product development. *Lancet Infect Dis.* **7**, 328-37 (2007)

Gutacker, M. M. *et al.* Genome-wide analysis of synonymous single nucleotide polymorphisms in Mycobacterium tuberculosis complex organisms: resolution of genetic relationships among closely related microbial strains. *Genetics* **162**, 1533-43 (2002)

Gutacker, M. M. *et al.* Single-nucleotide polymorphism-based population genetic analysis of Mycobacterium tuberculosis strains from 4 geographic sites. *J. Infect. Dis.* **193**, 121-8 (2006)

He, X. Y., Zhuang, Y. H., Zhang, X. G. & Li, G. L. Comparative proteome analysis of culture supernatant proteins of Mycobacterium tuberculosis H37Rv and H37Ra. *Microbes. Infect.* **5**, 851-6 (2003)

Hebert, A. M. *et al.* DNA polymorphisms in the pepA and PPE18 genes among clinical strains of Mycobacterium tuberculosis: implications for vaccine efficacy. *Infect. Immun.* **75**, 5798-805 (2007)

Hershberg, R. *et al.* High functional diversity in Mycobacterium tuberculosis driven by genetic drift and human demography. *PLoS Biol.* **6**, e311

Homolka, S., Köser, C., Archer, J., Rüsch-Gerdes, S. & Niemann, S. Single nucleotide polymorphisms in Rv2629 are specific for Mycobacterium tuberculosis genotypes Beijing and Ghana but not associated with rifampin resistance. *J. Clin. Microbiol.* **47**, 223-6 (2009)

Iwamoto, T., Yoshida, S., Suzuki, K. & Wada, T. Population structure analysis of the Mycobacterium tuberculosis Beijing family indicates an association between certain sublineages and multidrug resistance. *Antimicrob. Agents Chemother.* **52**, 3805-9 (2008)

Jiang, X. *et al.* Comparison of the proteome of isoniazid-resistant and -susceptible strains of Mycobacterium tuberculosis. *Microb. Drug Resist.* **12**, 231-8 (2006)

Jiang, X. *et al.* Identification of unique genetic markers in Rv0927c among Mycobacterium tuberculosis W-Beijing strains. *Microbes Infect.* **9**, 241-6 (2007)

Lari, N., Rindi, L., Bonanni, D., Tortoli, E. & Garzelli, C. Mutations in mutT genes of Mycobacterium tuberculosis isolates of Beijing genotype. *J. Med. Microbiol.* **55**, 599-603 (2006)

Lee, K. W., Lee, J. M. & Jung, K. S. Characterization of pncA mutations of pyrazinamide-resistant Mycobacterium tuberculosis in Korea. *J. Korean Med. Sci.* **16**, 537-43 (2001)

Lee, J. S. *et al.* Mutation in the transcriptional regulator PhoP contributes to avirulence of Mycobacterium tuberculosis H37Ra strain. *Cell Host Microbe* **14**, 97-103 (2008)

Liu, X., Gutacker, M. M., Musser, J.M. & Fu, Y. X. Evidence for recombination in Mycobacterium tuberculosis. *J. Bacteriol.* **188**, 8169-77 (2006)

Louw, G. E., Warren, R. M., van Helden, P. D. & Victor, T. C. Rv2629 191A/C nucleotide change is not associated with rifampicin resistance in Mycobacterium tuberculosis. *Clin. Chem. Lab. Med.* **54**, 542-3 (2009)

Musser, J. M., Amin, A. & Ramaswamy, S. Negligible genetic diversity of mycobacterium tuberculosis host immune system protein targets: evidence of limited selective pressure. *Genetics* **155**, 7-16 (2000)

Moreland, N. J., Charlier, C., Dingley, A. J., Baker, E. N. & Lott, J. S. Making Sense of a Missense Mutation: Characterization of MutT2, a Nudix Hydrolase from Mycobacterium tuberculosis , and the G58R Mutant Encoded in W-Beijing Strains of M. tuberculosis. *Biochemistry* **48**, 699-708 (2009)

Nouvel, L. X., Dos Vultos, T., Kassa-Kelembho, E., Rauzier, J. & Gicquel, B. A non-sense mutation in the putative anti-mutator gene ada/alkA of Mycobacterium tuberculosis and M. bovis isolates suggests convergent evolution. *BMC Microbiol.* **7**:39 (2007)

Okamoto, S. *et al.* Loss of a conserved 7-methylguanosine modification in 16S rRNA confers low-level streptomycin resistance in bacteria. *Mol. Microbiol.* **63**, 1096-106 (2007)

Olano, J. *et al.* Mutations in DNA repair genes are associated with the Haarlem lineage of Mycobacterium tuberculosis independently of their antibiotic resistance. *Tuberculosis (Edinb.)* **87**, 502-8 (2007)

Rad, M. E. *et al.* Mutations in putative mutator genes of Mycobacterium tuberculosis strains of the W-Beijing family. *Emerg. Infect. Dis.* **9**, 838-45 (2003)

Ramaswamy, S. V. *et al.* Molecular genetic analysis of nucleotide polymorphisms associated with ethambutol resistance in human isolates of Mycobacterium tuberculosis. *Antimicrob. Agents Chemother.* **44**, 326-36 (2000)

Ramaswamy, S. V. *et al.* Single nucleotide polymorphisms in genes associated with isoniazid resistance in Mycobacterium tuberculosis. *Antimicrob. Agents Chemother.* **47**, 1241-50 (2003)

Rindi, L. *et al.* Variation of the expression of Mycobacterium tuberculosis ppe44 gene among clinical isolates. *FEMS Immunol. Med. Microbiol.* **51**, 381-7 (2007)

Rindi, L., Lari, N., Cuccu, B. & Garzelli, C. Evolutionary pathway of the Beijing lineage of Mycobacterium tuberculosis based on genomic deletions and mutT genes polymorphisms. *Infect. Genet. Evol.* Epub ahead of print (2008)

Safi, H., Sayers, B., Hazbón, M.H. & Alland, D. Transfer of embB codon 306 mutations into clinical Mycobacterium tuberculosis strains alters susceptibility to ethambutol, isoniazid, and rifampin. *Antimicrob Agents Chemother.* **52**, 2027-34 (2008)

Sekiguchi, J. *et al.* Detection of multidrug resistance in Mycobacterium tuberculosis. *J. Clin. Microbiol.* **45**, 179-92 (2007)

Siddiqi, N. *et al.* Molecular characterization of multidrug-resistant isolates of Mycobacterium tuberculosis from patients in North India. *Antimicrob Agents Chemother.* **46**, 443-50 (2002)

Spies, F. S., Almeida da Silva, P. E., Ribeiro, M. O., Rossetti, M. L. & Zaha, A. Identification of mutations related to streptomycin resistance in clinical isolates of Mycobacterium tuberculosis and possible involvement of efflux mechanism. *Antimicrob. Agents Chemother.* **52**, 2947-9 (2008)

Sreevatsan, S. *et al.* Restricted structural gene polymorphism in the Mycobacterium tuberculosis complex indicates evolutionarily recent global dissemination. *Proc. Natl. Acad. Sci. U S A* **94**, 9869-74 (1997)

van Doorn, H. R. *et al.* The susceptibility of Mycobacterium tuberculosis to isoniazid and the Arg→Leu mutation at codon 463 of katG are not associated. *J. Clin. Microbiol.* **39**, 1591-4 (2001)

Wang, Q. *et al.* A Newly Identified 191A/C Mutation in the Rv2629 Gene that Was Significantly Associated with Rifampin Resistance in Mycobacterium tuberculosis. *J. Proteome Res.* **6**, 4564-71 (2007)

Zheng, H. *et al.* Genetic Basis of Virulence Attenuation Revealed by Comparative Genomic Analysis of Mycobacterium tuberculosis Strain H37Ra versus H37Rv. *PLoS ONE* **3**, e2375

2 Supplementary Methods

2.1 SNPs

Our data is presented in Excel format (File S2) so that they are widely accessible for further studies. The file contains 5 worksheets, each of which corresponds to one of sections of the Venn diagram (Figure 2). SNPs that were present in all three genomes ("common to all 3 genomes" worksheet) and were found to be errors in the original H37Rv sequence (Supplementary Table 1) are highlighted in blue.

Using the filtering function (grey box with an upside-down pyramid) activated in the first line of each worksheet, the data can be easily sorted according to the entries available. Provided macros are activated, entries in the columns "gene name", "UniProt", and "EC number" will function as HTTP links. Furthermore, the "accession numbers" provide links to the Kyoto Encyclopedia of Genes and Genomes[1].

The annotation of the H37Rv genome (AL123456.2) was obtained from TubercuList[2].

In addition, the following columns are provided:

2.1.1 Position of change

Base pair position of SNP relative to H37Rv. If a change affected two overlapping genes, the appropriate changes are shown in consecutive lines.

2.1.2 Score

The scores of the base call come from our in-house Bayesian allele caller. This takes into account base calls and (phred-equivalent) quality values. A score of 12 is roughly equivalent to four Q30 base calls.

Where a SNP occurred in more than one isolate the lowest score is shown.

2.1.3 Base change

Base changes are separated by "/" where the first entry refers to H37Rv and the second to the new genome. The affected bases are CAPITALSED. The triplet is shown if a SNP fell within a protein coding sequence. Where adjacent SNPs affected the same amino acid, the CAPITALISED base in question is underlined.

For inter-genic SNPs, the base on the forward strand is shown.

2.1.4 Triplet position

Triplet codon positions are 1, 2 or 3 for the forward strand and -1, -2 or -3 for the reverse strand. Positions are not given in case of non-coding inter-genic, tRNA or rRNA changes.

[1] Kanehisa, M. *et al.* KEGG for linking genomes to life and the environment. *Nucleic Acids Res.* **36**, D480-D484 (2008) http://www.genome.jp/kegg/

[2] Cole, S. T. Learning from the genome sequence of Mycobacterium tuberculosis H37Rv. *FEBS Lett.* **452**, 7-10 (1999) TubercuList, release 11

2.1.5 (Amino acid) Change

Inter-genic The distance in base pairs to the two genes located nearest on either side of the SNP (Section 2.1.7) is given.

Intra-genic SNPs (excluding tRNA and rRNA), the amino acids were determined using the bacterial genetic code[1]. (s) highlights the potential for a start codon. The number denotes the amino acid position of the change relative to the start of the gene in H37Rv.

2.1.6 Comment I

Entries include inter-genic SNPs, tRNA SNP and rRNA SNP. Any changes within protein coding sequences which do not change the amino acid (irrespective of potential to act as a start codon) are synonymous (sny). Otherwise, they are non-synonymous (non-syn).

2.1.7 Comment II

Inter-genic The names of the two genes located nearest on either side of the SNP are given (Section 2.1.5).

Intra-genic Using a Blosum62 table[2], non-synonymous changes which had a 0 or positive value associated were categorised as conservative (cons). Otherwise, they are non-conservative (non-cons). All non-synonymous changes involving stop codons were treated as non-conservative.

With the exception of “location (kb)”, “calculated map” and “codon usage” all the “gene list columns” were included.

[1] http://www.ncbi.nlm.nih.gov/Taxonomy/Utils/wprintgc.cgi?mode=t#SG11 accessed 18.10.2007

[2] Henikoff, S. & Henikoff, J. G. Amino acid substitution matrices from protein blocks. *Proc. Natl. Acad. Sci. U S A* **89**, 10915-9 (1992)
http://expasy.org/cgi-bin/blosum.pl

2.1.8 Operon 1-5

Polar effects may be caused by non-synonymous SNPs that either abolish a start codon or introduce a premature stop codon. To allow the reader to assess such events, we considered whether a change was located within an experimentally confirmed (cR) or predicted (pR) operon[1]. If both cases applied, both (bR) is displayed in column "operon 1". The entries in operon 2-5 are based on alternative algorithms to predict operons. Entries include pM[2], pRa[3], pP[4] and pW[5].

2.1.9 Nature of genes affected

Genes were classified as essential (ess) and/or non-essential (non-ess) according to Lamichhane[6], McAdam[7], Sassetti[8] and Yesilkaya[9].

Please note that for some genes the information may be contradictory or not available at all.

[1]Roback, P. *et al.* A predicted operon map for Mycobacterium tuberculosis. *Nucleic Acids Res.* **35**, 5085-95 (2007)
Rv2232 replaced *Rv2232* and *Rv2233* in previous releases. Therefore, operon *Rv2233-Rv2235* was treated as *Rv2232-Rv2235*.

[2]Moreno-Hagelsieb, G. & Collado-Vides, J. A powerful non-homology method for the prediction of operons in prokaryotes. *Bioinformatics* 2002;18 Suppl 1:S329-36 (2002)
Reddy, T. B. K. *et al.* TB database: an integrated platform for tuberculosis research. *Nucleic Acids Res.* (2008)
http://www.broad.mit.edu/annotation/genome/tbdb/OperonSearchResults.html

[3]Ranjan, S., Gundu, R. K. & Ranjan, A. MycoperonDB: a database of computationally identified operons and transcriptional units in Mycobacteria. *BMC Bioinformatics* **7**, S9 (2006)

[4]Ptools-OFS[a]

[5]WUStL-OFS[a]

[6]Lamichhane, G. *et al.* A postgenomic method for predicting essential genes at subsaturation levels of mutagenesis: application to Mycobacterium tuberculosis. *Proc. Natl. Acad. Sci. U S A* **100**, 7213-8 (2003)

[7]McAdam, R. A. *et al.* Characterization of a Mycobacterium tuberculosis H37Rv transposon library reveals insertions in 351 ORFs and mutants with altered virulence. *Microbiology* **148**, 2975-86 (2002)

[8]Sassetti, C. M., Boyd, D. H. & Rubin, E. J. Genes required for mycobacterial growth defined by high density mutagenesis. *Mol. Microbiol.* **48**, 77-84 (2003)

[9]Yesilkaya, H., Dale, J. W., Strachan, N. J. & Forbes, K. J. Natural transposon mutagenesis of clinical isolates of Mycobacterium tuberculosis: how many genes does a pathogen need? *J. Bacteriol.* **187**, 6726-32 (2005)

[a]Squires, B. *et al.* BioHealthBase: informatics support in the elucidation of influenza virus host pathogen interactions and virulence. *Nucleic Acids Res.* **36**, D497-503 (2008)
A detailed description of the protocols used to predict these operons can be found online at the BioHealthBase.
Our thanks to Shubhada Godbole (U.T. Southwestern Medical Center) and Christopher Larsen (Vecna Technologies) for sharing this data.

2.1.10 SNPs across genomes

In order to facilitate a comparison to previous large scale studies[1] and to facilitate further study of the SNPs determined in this study we computed SNPs in a number of closely related mycobacterial genomes[2] relative to H37Rv. This was done using MUMmer 3.20 with its nucmer and show-snps functions.[3] We removed SNPs from the above alignment which were located within repeat areas with an identity > 90% to exclude false SNPs that might have been caused by poor alignment of the repetitive regions rather than being true polymorphism in homologous regions.[4] The presence of a SNP is shown by an "x". Please note that this comparison was done on the nucleotide and *not* the amino acid level. Given the degeneracy of the triplet code it is possible for a different nucleotide change within a codon to yield the same amino acid change.

---

[1] The following studies are based on comparisons of H37Rv to 210, CDC1551 or *M. bovis*:

Gutacker, M. M. *et al.* Genome-wide analysis of synonymous single nucleotide polymorphisms in Mycobacterium tuberculosis complex organisms: resolution of genetic relationships among closely related microbial strains. *Genetics* **162**, 1533-43 (2002)

Alland, D. *et al.* Modeling bacterial evolution with comparative-genome-based marker systems: application to Mycobacterium tuberculosis evolution and pathogenesis. *J. Bacteriol.* **185**, 3392-9 (2003)

Gutacker, M. M. *et al.* Single-nucleotide polymorphism-based population genetic analysis of Mycobacterium tuberculosis strains from 4 geographic sites. *J. Infect. Dis.* **193**, 121-8 (2006)

Liu, X., Gutacker, M. M., Musser, J.M. & Fu, Y. X. Evidence for recombination in Mycobacterium tuberculosis. *J. Bacteriol.* **188**, 8169-77 (2006)

[2] *M. tuberculosis*

complete: CDC1551 (AE000516.2), F11 (CP000717.1) and H37Ra (CP000611.1)

partial: 210[a]

M. bovis

complete: AF2122/97 (BX248333.1) and BCG str. Pasteur 1173P2 (AM408590.1)

[3] Kurtz, S. *et al.* Versatile and open software for comparing large genomes. *Genome Biol.* **5**, R12 (2004)

[4] The following commands were used:

1. `nucmer --prefix=ref_qry ref.fasta qry.fasta`
2. `show-snps -ClrI ref_qry.delta > ref_qry.snps`

Repeats in the H37Rv genome were determined using:

3. `nucmer --maxmatch --nosimplify --prefix=seq_seq seq.fasta seq.fasta`
4. `show-coords -r seq_seq.delta > seq_seq.coords`

For a detailed manual please refer to `http://mummer.sourceforge.net/manual/`

Our thanks to Kathryn Holt (Wellcome Trust Sanger Institute) for her help with the alignment.

[a] Barnes, P. F. *et al.* Patterns of tuberculosis transmission in Central Los Angeles. *JAMA* **278**, 1159-63 (1997)

2.2 Graphical display of results

2.2.1 Opening the files

We provided a graphical way of displaying our SNP results using the Artemis[1] genome viewer[2]. It was designed to be independent of external databases to preserve both the version of the genome and annotation used for this study, both of which are likely be subject to change in the near future. A detailed manual[3] and helpful demo[4] of Artemis are available online. Here instructions aimed at the generalist are appended:

1. A UNIX, GNU/Linux, BSD, Macintosh and MS Windows compatible version of Artemis can be obtained from `http://www.sanger.ac.uk/Software/Artemis/v11/`. Do **not** use the "LAUNCH ARTEMIS" button but the appropriate "Alternatively" link underneath to ensure that you download "artemis_v11.jar" (**not** "Artemis.jnlp") to a folder of your choice[5].

2. Next go to the appropriate folder containing "artemis_v11.jar" and right-click on the "artemis_v11.jar" icon to execute the "Create Shortcut" command. Then right-click on the shortcut just created and add "java -mx800m -jar " (make sure to include a space after "-jar") the start of the "Target:" field (please note that Windows may replace "java" with the location of the appropriate executable which is fine). This will set the maximum memory to 800 MB which should suffice to open multiple graphs. For more details about increasing the memory in general and specific instructions for UNIX or GNU/Linux users refer to `http://www.sanger.ac.uk/Software/Artemis/faqs.shtml`.

3. Once the programm is launched using the shortcut (do **not** use "artemis_v11.jar"), change the "Genetic Codes Tables" in the "Options" menu from "1. Standard" to "11. Bacterial and Plant Plastid".

4. Open the annotated *M. tuberculosis* H37Rv genome file[6] ("AL123456_2_TubercuList_R11.embl" (File S3)) using the "Open..." command on the "File" menu.

5. Use "Graph → Add User Plot" and select one of the graph files ("H37Rv.txt" (File S4), "K-1.txt" (File S5) or "K-2.txt" (File S6)). After a few seconds (please be patient), a small window with the corresponding graph should appear just above the genome (refer to Supplementary Figure 1). If not, a restart of the programme becomes necessary. Should the problem persist, it may be because your computer has insufficient memory (refer to point 4. at `http://www.sanger.ac.uk/Software/Artemis/faqs.shtml`).

6. Right-click on the graph and "Set the Window Size" to 1.

7. To zoom in and out of the genome, use the scrollbars at the right-hand side of the different

[1] Rutherford, K. *et al.* Artemis: sequence visualization and annotation. *Bioinformatics* **16**, 944-5 (2000)

[2] Our thanks to Arnab Pain from the European Bioinformatics Institute and Kim Rutherford from FlyMine (University of Cambridge) for their help with Artemis.

[3] `http://www.sanger.ac.uk/Software/Artemis/v11/manual/`

[4] `http://www.sanger.ac.uk/Software/Artemis/demo/artemis1.htm`

[5] Internet Explorer may name the downloaded file "artemis_v11.zip". Be sure to rename it "artemis_v11.jar" before following any of the other instructions.

[6] AL123456.2 and TubercuList, release 11

sub-windows. “Goto $\rightarrow$ Navigator $\rightarrow$ Goto Base” allows you to jump to any position within the genome.

2.2.2 The output

Genome The genome is displayed in the bottom three sections of the window. Each gene contains a small summary of the information from TubercuList (gene name, a note of its function and class[1] that can be viewed by clicking onto a gene followed by "Ctrl+E", refer to Supplementary Figure 1). Here is the key of the output scheme.

FUNCTIONAL AND COLOUR CLASSIFICATION[2]

0	Virulence, detoxification, adaptation
1	Lipid metabolism
2	Information pathways
3	Cell-wall and cell processes
4	Stable RNAs
5	Insertion sequences and phages
6	PE and PPE proteins
7	Intermediary metabolism and respiration
8	Unknown[3]
9	Regulatory proteins
10	Conserved hypotheticals[4]
16	Conserved hypotheticals with an orthologue in *M. bovis*[5]

SNPs The remaining three genome files contain their respective SNPs. The colour of the peaks (Supplementary Figure 1) codes for the location of a SNP within the Venn diagram (Figure 2). The height of the peaks describes the nature of the SNP.

Intersections

SPECIFIC TO THE GENOME IN QUESTION

BEIJING K-FAMILY BACKBONE

COMMON TO ALL 3 GENOMES

Description

PEAK-HEIGHT 4

non-synonymous or *inter-genic*

PEAK-HEIGHT 2

synonymous

PEAK-HEIGHT 3

synonymous in one gene but *non-synonymous in a second, overlapping gene*

---

[1]Please note that if a gene was assigned to multiple classes, only the first class is shown in this representation with the appropriate colour coding.

[2]Cole, S. T. Learning from the genome sequence of Mycobacterium tuberculosis H37Rv. *FEBS Lett.* **452**, 7-10 (1999) TubercuList, release 11

[3]Unknown genes found in *M. tuberculosis* only.

[4]Unknown function but found in other species.

[5]Genes of unknown function found in *M. tuberculosis* and *M. bovis*, exclusively.

3 Supplementary Results

3.1 Drug resistance

All the mutation detected in this study, except the one in *pncA*, are well established to confer resistance. In fact, the nonsense mutation at amino acid 119 was previously described in both a sensitive and resistant isolate[1]. However, given that PZA is a prodrug that is converted to its active form, pyrazinoic acid, by *pncA*, this premature stop codon should be responsible for resistance[2].

[1]Lee, K. W., Lee, J. M. & Jung, K. S. Characterization of pncA mutations of pyrazinamide-resistant Mycobacterium tuberculosis in Korea. *J. Korean Med. Sci.* **16**, 537-43 (2001)
Chan, R.C. *et al.* Genetic and phenotypic characterization of drug-resistant Mycobacterium tuberculosis isolates in Hong Kong. *J. Antimicrob. Chemother.* **59**, 866-873 (2007)

[2]Scorpio, A. & Zhang, Y. Mutations in pncA, a gene encoding pyrazinamidase/nicotinamidase, cause resistance to the antituberculous drug pyrazinamide in tubercle bacillus. *Nat. Med.* **2**, 662-667 (1996)
